# Estrogen receptor activation contributes to RNF146 expression and neuroprotection in Parkinson's disease models

**DOI:** 10.18632/oncotarget.21828

**Published:** 2017-10-11

**Authors:** Hyojung Kim, Sangwoo Ham, Joon Yeop Lee, Areum Jo, Gum Hwa Lee, Yun-Song Lee, MyoungLae Cho, Heung-Mook Shin, Donghoon Kim, Olga Pletnikova, Juan C. Troncoso, Joo-Ho Shin, Yun-Il Lee, Yunjong Lee

**Affiliations:** ^1^ Division of Pharmacology, Department of Molecular Cell Biology, Sungkyunkwan University School of Medicine, Samsung Biomedical Research Institute, Suwon 440-746, Republic of Korea; ^2^ National Development Institute of Korean Medicine, Gyeongsan 38540, Republic of Korea; ^3^ College of Pharmacy, Chosun University, Gwangju 501-759, Republic of Korea; ^4^ Department of Neurology, The Johns Hopkins University School of Medicine, Baltimore, Maryland, USA; ^5^ Department of Pathology, Division of Neuropathology, The Johns Hopkins University School of Medicine, Baltimore, Maryland, USA; ^6^ Single Cell Network Research Center, Sungkyunkwan University School of Medicine, Suwon, Gyeonggi-Do 440-746, Republic of Korea; ^7^ Well Aging Research Center, Daegu Geongbuk Institute of Science and Technology, Daegu 42988, South Korea; ^8^ Companion Diagnostics and Medical Technology Research Group, Daegu Geongbuk Institute of Science and Technology, Daegu 42988, South Korea

**Keywords:** luciferase screen, preconditioning, RNF146, AIMP2, PARP1-dependent cell death

## Abstract

RNF146 is an E3 ubiquitin ligase that specifically recognizes and polyubiquitinates poly (ADP-ribose) (PAR)-conjugated substrates for proteasomal degradation. RNF146 has been shown to be neuroprotective against PAR polymerase-1 (PARP1)-induced cell death during stroke. Here we report that RNF146 expression and RNF146 inducers can prevent cell death elicited by Parkinson’s disease (PD)-associated and PARP1-activating stimuli. In SH-SY5Y cells, RNF146 expression conferred resistance to toxic stimuli that lead to PARP1 activation. High-throughput screen using a luciferase construct harboring the RNF146 promoter identified liquiritigenin as an RNF146 inducer. We found that RNF146 expression by liquiritigenin was mediated by estrogen receptor activation and contributed to cytoprotective effect of liquiritigenin. Finally, RNF146 expression by liquiritigenin in mouse brains provided dopaminergic neuroprotection in a 6-hydroxydopamine PD mouse model. Given the presence of PARP1 activity and RNF146 deficits in PD, it could be a potential therapeutic strategy to restore RNF146 expression by natural compounds or estrogen receptor activation.

## INTRODUCTION

RNF146 is an E3 ubiquitin ligase that recognizes its substrates via poly (ADP-ribose) (PAR). The ligase activity of RNF146 is stimulated upon binding to PAR through conformational changes [1–3]. RNF146 polyubiquitinates protein substrates that are covalently conjugated to PAR or that noncovalently interact with PAR and targets them for proteasomal degradation [2, 4, 5]. Depending on the substrate, RNF146 expression in combination with PAR modification of substrates influences diverse cellular functions [6–8]. Selective recognition of RNF146 substrates is mainly determined by PARsylation of proteins, which is predominantly mediated by poly (ADP-ribose) polymerases (PARPs). The PARP superfamily has 17 members, among which PARP1 performs the majority of the poly (ADP-ribose) synthesis [9, 10]. Interestingly, PARP1 itself serves as a substrate for RNF146 when PARP1 is PARsylated upon the appropriate signals. PARP1 activation, PARsylation of PARP1 substrates, and PAR polymers play roles in diverse cellular functions including genomic stability maintenance, transcriptional regulation, cell proliferation, and cell death [8, 10]. As an antagonizing modulator of PARP1, RNF146 can affect PARP1-regulated cellular processes. This regulation depends on the cellular context, which determines the roles of PARP1 and its substrates [8, 10]. For example, RNF146 has been shown to be neuroprotective against DNA damage, N-methyl-D-aspartate (NMDA)-induced excitotoxicity, and stroke, all of which induce neuronal damage via overactivation of PARP1 enzymatic activity and PAR polymer synthesis [6]. The pathological form of cell death involving PARP1 overactivation was termed parthanatos [9]. Although parthanatos has also been reported to be involved in various neurodegenerative diseases including PD [11, 12], the therapeutic potential of RNF146 expression in the context of PD has not been evaluated.

Several studies in PD mouse models have supported the involvement of PARP1 activity in progressive dopaminergic neuronal loss [11, 13, 14]. For example, mitochondrial 1-methyl-4-phenyl-1,2,3,6-tetrahydropyridine (MPTP) intoxication leads to PARP1 overactivation in dopaminergic neurons via excitotoxicity and oxidative radical-induced DNA damage, a strong stimulator of PARP1 activity [13]. Moreover, conditional transgenic mice expressing the parkin substrate aminoacyl tRNA synthetase complex-interacting multifunctional protein 2 (AIMP2) also display progressive loss of dopaminergic neurons [11]. AIMP2-mediated activation of PARP1 is largely responsible for dopamine (DA) neuron loss. This conclusion is supported by evidence showing that PARP1 inhibitor treatment or PARP1-knockout prevents DA neuron loss [11]. Although an L-DOPA regimen is prescribed to supplement the reduced level of the neurotransmitter dopamine in PD as an antisymptomatic measure, there is an unmet need to develop therapeutic strategies that can fundamentally slow or halt ongoing neurodegeneration in PD. In this respect, compounds that can modulate PARP1 activity, either directly or indirectly, have the potential to benefit patients suffering from acute neuronal damage and patients suffering from slowly progressing neurodegenerative diseases.

RNF146 is present in low levels in brain tissue [6]. Moreover, increasing the RNF146 content in neurons has been shown to confer resistance to PARP1-stimulated cell death. Indeed, RNF146 was initially identified as an NMDA-induced plasticity late response gene [15]. Low-dose 1-methyl-3-nitro-1-nitrosoguanidine (MNNG), low-dose NMDA, and short exposure to oxygen glucose deprivation all induce the expression of multiple genes in neurons, some of which provide resistance against subsequent strong cell death stimuli [15]. RNF146 expression is substantially induced in these sublethal dose preconditioning procedures; however, the molecular pathways underlying these processes are unknown [6, 15]. Since it is not practical to perform low-dose toxin preconditioning, screening of safe natural compounds that induce RNF146 expression has the potential to identify therapeutic lead compounds for further development to prevent PARP1 activation and PD pathogenesis. In addition to PARP1 inhibition and prevention of neuronal cell death, RNF146 has been shown to regulate cell survival and proliferation via Wnt/beta-catenin [5, 16] or PTEN-Akt [4] pathways. Collectively, RNF146 regulation could be beneficial in preventing main cell death pathways including parthanatos and apoptosis.

Here we show for the first time that RNF146 expression is indeed sufficient to prevent PARP1-dependent cell toxicity in neuroblastoma PD model cell lines. We established an RNF146 luciferase reporter system to monitor RNF146 promoter activity and screened several RNF146-inducing natural compounds. Among the compounds screened was liquiritigenin, an active estrogenic compound from the root of *Glycyrrhizae uralensis*. RNF146 induction by liquiritigenin is mediated via estrogen receptor activation. Liquiritigenin induction of RNF146 inhibits PARP1 and prevents cell death in *in vitro* and *in vivo* PD models, suggesting the therapeutic potential of RNF146-inducing compounds in PD.

## RESULTS

### RNF146 expression prevents PARP1-dependent cell death in PD model cell lines

**Figure 1:**
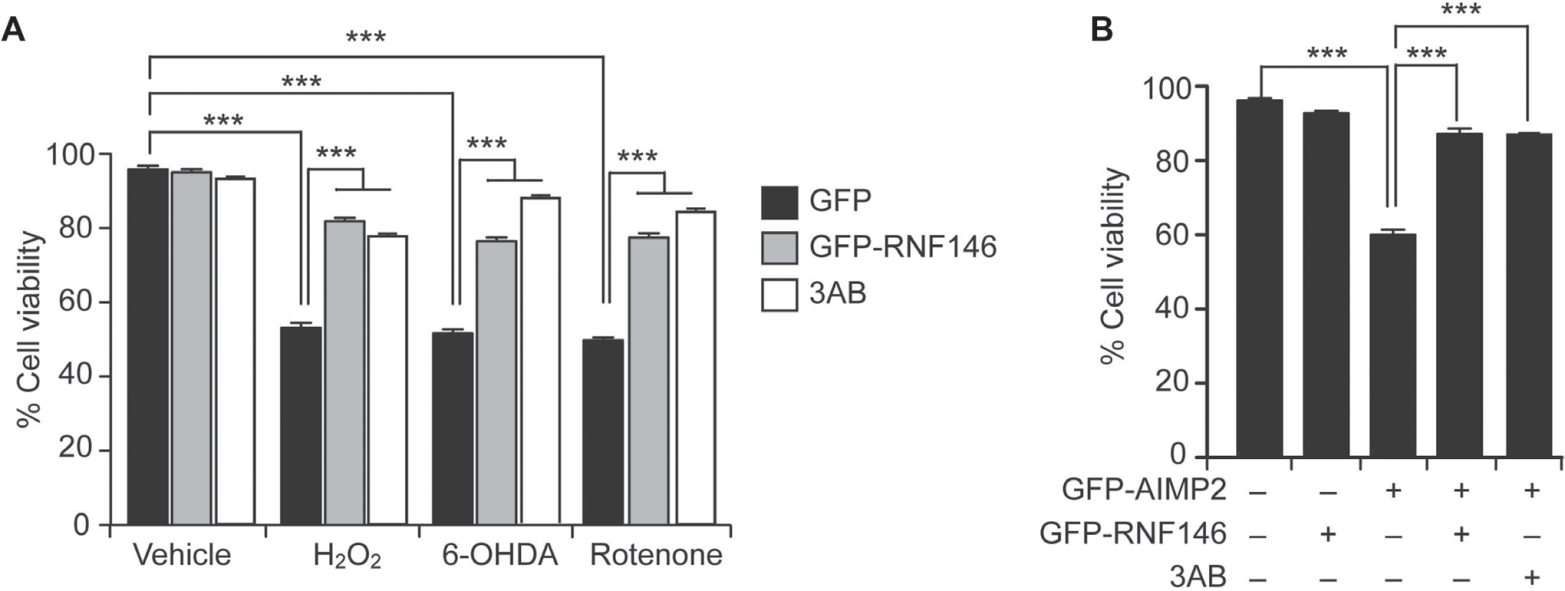
RNF146 expression prevents PARP-1-dependent cell death in SH-SY5Y cells (**A**) Trypan blue exclusion cell viability assay demonstrating that GFP-RNF146 expression and PARP inhibition both increase cell survival after stimulation with H_2_O_2_ (1 mM), 6-OHDA (70 uM), or rotenone (20 uM). SH-SY5Y cells were transfected with constructs driving the expression of GFP or GFP-RNF146 for 24 hrs and then treated for 24 hrs with each toxin at the indicated concentration. The PARP inhibitor 3AB (10 uM) was added to SH-SY5Y cells 4 hrs before toxin treatment (*n* = 6). (**B**) Trypan blue exclusion viability assay demonstrating that GFP-RNF146 coexpression (30 hrs) or 3AB (10 uM) treatment confers complete protection against GFP-AIMP2-mediated toxicity in SH-SY5Y cells (*n* = 5). Data are expressed as mean ± SEM. ^***^P < 0.001, ANOVA test followed by Tukey post hoc analysis.

RNF146 expression suppresses PARP1 activation and cell death upon DNA damage, NMDA excitotoxicity, and oxygen glucose deprivation [6, 15]. Since PARP1 activation has been reported in various neurodegenerative models including PD, we evaluated the therapeutic potential of RNF146 expression in neuroblastoma SH-SY5Y cells challenged with PD-related toxins. Consistent with previous reports on hydrogen peroxide (H_2_O_2_)-induced PARP1 activation and cell toxicity [11], 1 mM H_2_O_2_ treatment reduced cell viability by 50%. This effect was largely prevented by PARP inhibitor 3-aminobenzamide (3AB) treatment or by expression of RNF146 (Figure 1A and Supplementary Figure 1A). Of note, RNF146 expression was approximately as protective against H_2_O_2_-mediated toxicity as 3AB. Treatment with other toxins (e.g., 6-hydroxydopamine (6-OHDA), and rotenone) commonly used to model PD in cells [17, 18] resulted in dose-dependent reductions in the viability of SH-SY5Y cells, as determined by the trypan blue exclusion assay (Supplementary Figure 1B, 1C). Similar to the H_2_O_2_ results, both RNF146 expression and PARP1 inhibition prevented the majority of the cell death induced by 6-OHDA or rotenone treatment (Figure 1A). Recently, expression of the parkin substrate AIMP2 was shown to stimulate PARP1 activation and parthanatos (PARP1-dependent cell death paradigm) [11]. Abnormal AIMP2 accumulation leads to direct binding and hyperactivation of PARP1 [11], thus AIMP2 overexpression in cells can serve as an ideal PD genetic model of parthanatos. To determine whether RNF146 expression is cytoprotective in genetic PD cell models, SH-SY5Y cells were transfected with GFP-AIMP2, either with or without GFP-RNF146 coexpression (Supplementary Figure 1D). AIMP2 expression decreased the cell viability to approximately 60% that of control cells; this decrease was mostly prevented by either RNF146 coexpression or PARP inhibitor treatment (Figure 1D). These results indicate that RNF146 expression could help prevent PARP1-dependent cell toxicity in PD-associated toxin-induced cell models.

### Establishment of an RNF146 luciferase reporter construct

**Figure 2:**
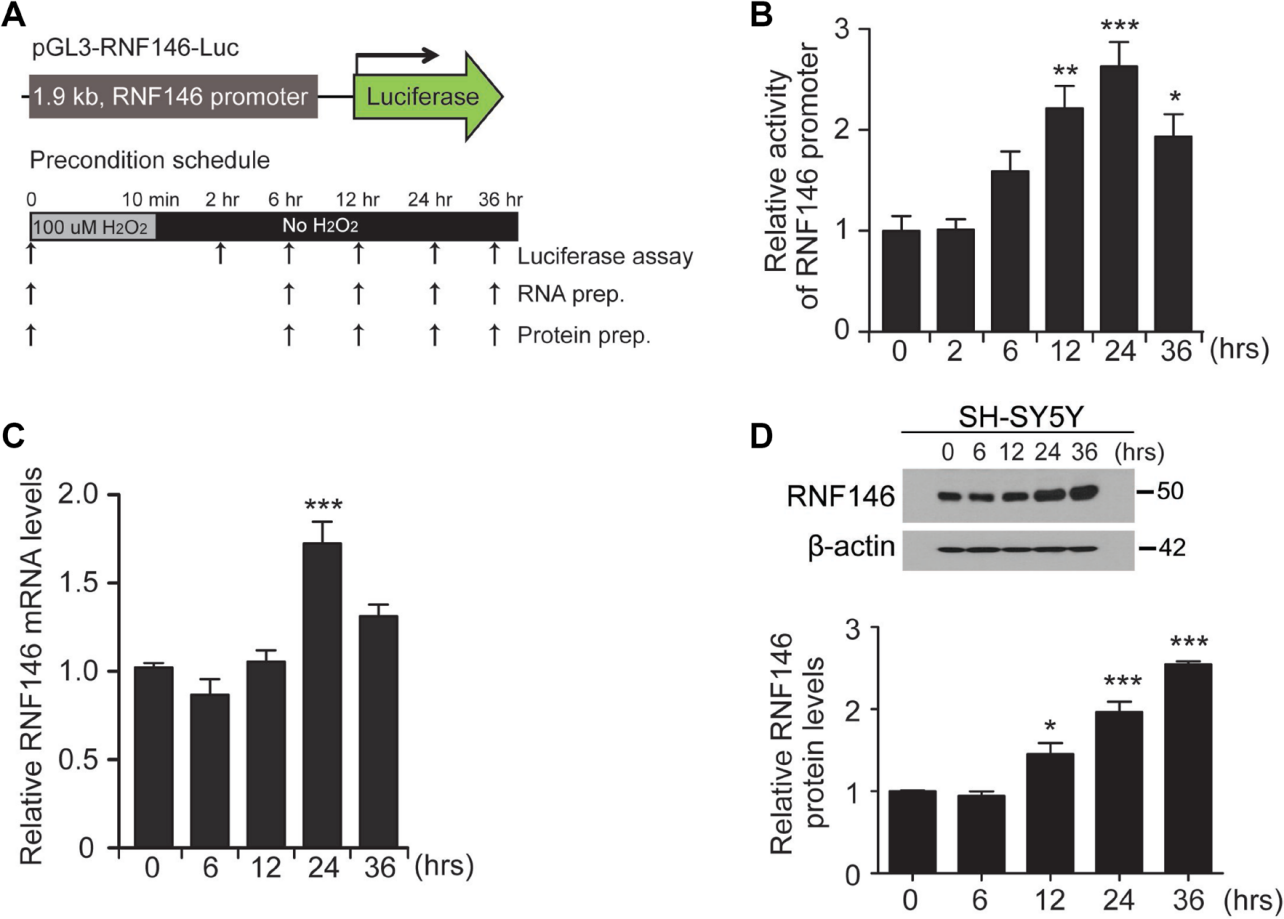
Generation of the RNF146 reporter construct (**A**) Schematic diagram of the luciferase construct with the 1.9 kb human RNF146 promoter (pGL3-RNF146-Luc). The experimental schedule for H_2_O_2_ preconditioning is illustrated in the bottom panel. SH-SY5Y cells were treated with 100 uM H_2_O_2_ for 10 min, washed briefly, and maintained in complete medium. Luciferase assay, total RNA isolation, or protein extract preparation was performed at the indicated time points. (**B**) Relative RNF146 promoter activities in SH-SY5Y cells at the indicated time points following preconditioning (100 uM H_2_O_2_,10 min), as determined by the luciferase assay (*n* = 3). SH-SY5Y cells were cotransfected with pGL3-RNF146-Luc and pRL-TK for 24 hrs, followed by low-dose H_2_O_2_ preconditioning. (**C**) Quantification by RT-qPCR of relative RNF146 messenger RNA levels (normalized to GAPDH) in SH-SY5Y cells at the indicated time points after low-dose H_2_O_2_ preconditioning (100 uM H_2_O_2_,10 min, *n* = 3). (**D**) Representative western blot showing RNF146 expression in SH-SY5Y cells at the indicated time points following H_2_O_2_ preconditioning (100 uM H_2_O_2_,10 min). β-actin serves as a loading control. Quantification of relative RNF146 protein levels normalized to those of β-actin at the indicated time points after H_2_O_2_ preconditioning (*n* = 3). Data are expressed as mean ± SEM. ^*^P < 0.05, ^**^P < 0.01, and ^***^P < 0.001, ANOVA test followed by Tukey post hoc analysis.

Based on the toxicity results in PD cell models, we hypothesized that RNF146-inducing compounds are neuroprotective against PD-associated toxins and in PD pathogenesis. Therefore, we established a screen for RNF146-inducing compounds. For high throughput compound screening, we constructed luciferase constructs harboring the human RNF146 promoter region (approximately 1.9 kb in length) into the pGL3 backbone (pGL3-RNF146-Luc) (Figure 2A). To determine whether this reporter construct reflects RNF146 expression in a similar manner as endogenous RNF146, we first tested this construct in a preconditioning low-dose hydrogen peroxide treatment protocol (Figure 2A). As shown in several low-dose toxin preconditioning studies [15], a 10 min treatment of low-dose hydrogen peroxide (100 uM) conferred resistance of SH-SY5Y cells to subsequent toxic concentrations of hydrogen peroxide treatment (Supplementary Figure 2A, 2B). By using this cell plasticity induction protocol with low-dose H_2_O_2_, we monitored RNF146 promoter activity by cotransfecting SH-SY5Y cells with pGL3-RNF146-Luc and pRL-TK (the latter was used for transfection normalization). RNF146 promoter activity gradually increased up to approximately 2.5-fold at 24 h compared to the level at 0 h (Figure 2B). RNF146 promoter activity was slightly decreased at 36 hrs but was still greater than that of the 0 hr control (Figure 2B). Mirroring the RNF146 promoter-driven luciferase activity, the RNF146 mRNA and protein levels were also elevated at 24 hr and 36 hrs compared to their corresponding levels in the 0 hr control (Figure 2C, 2D). Collectively, these data led us to conclude that pGL3-RNF146-Luc reflected RNF146 mRNA and protein induction and could therefore be suitably employed to screen for natural compounds that increase RNF146 expression.

### High-throughput natural compound screening identifies RNF146 inducers

**Figure 3:**
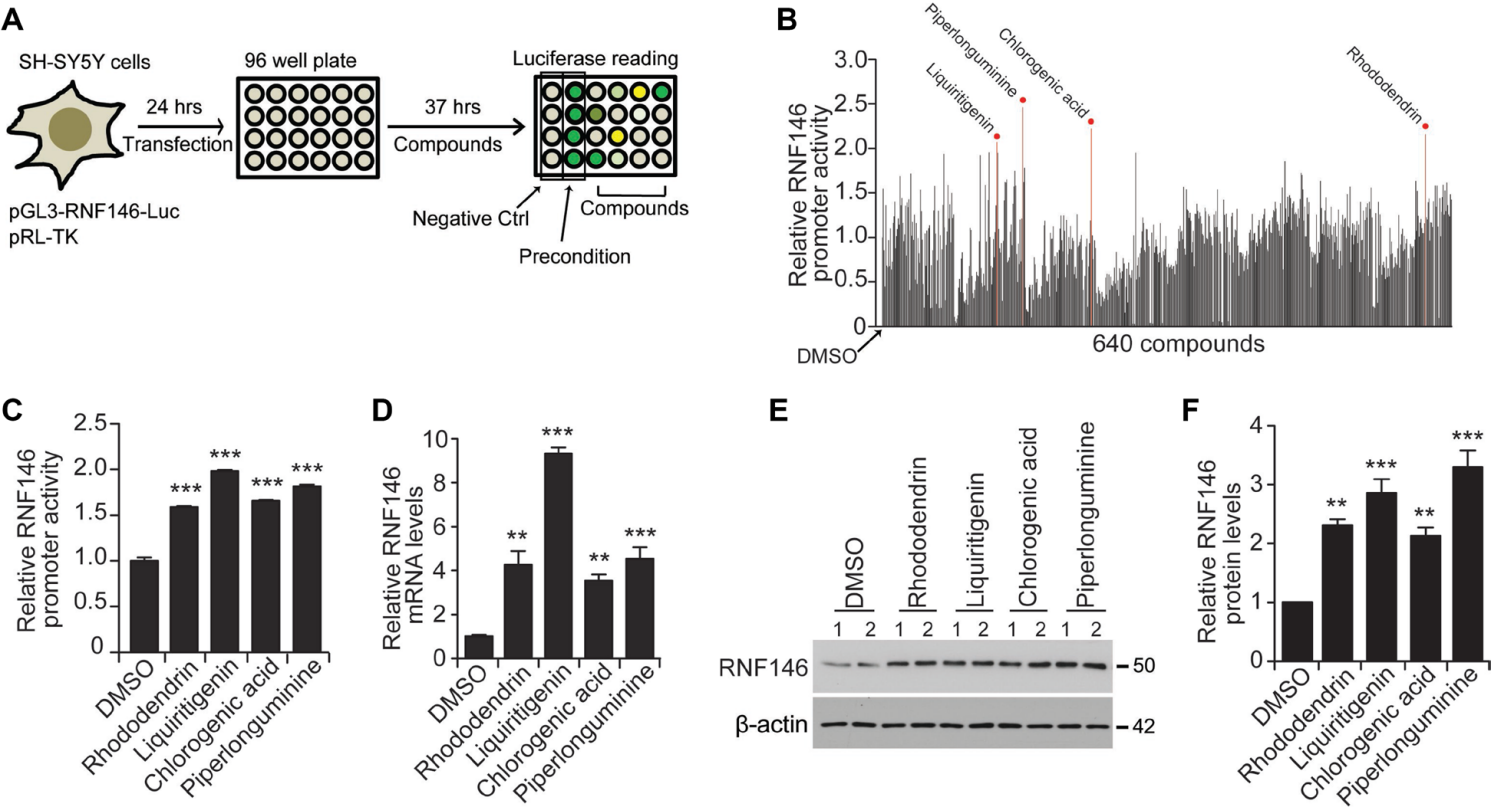
Screen for RNF146-inducing natural compounds (**A**) Schematic summary of the high-throughput natural compound (library of 640 individual compounds) screen. The screen employed a dual luciferase assay in SH-SY5Y cells transfected with the RNF146 promoter luciferase construct and pRL-TK. DMSO treatment and H_2_O_2_ preconditioning were used as negative and positive controls, respectively. (**B**) Relative RNF146 promoter activities in SH-SY5Y cells transfected with pGL3-RNF146-Luc and pRL-TK and then treated with the 640 natural compounds. Activities were determined by the luciferase assay. Firefly luciferase activity was normalized to that of *Renilla* luciferase. The normalized luciferase values are expressed relative to that of the DMSO negative control. Top four compounds that activated RNF146 promoter are indicated. (**C**) Quantification of relative RNF146 promoter activities in SH-SY5Y cells transfected with pGL3-RNF146-Luc and pRL-TK for 24 hrs, followed by 37 hrs treatment with 10 uM of the following RNF146-inducing compounds: rhododendrin, liquiritigenin, chlorogenic acid, and piperlonguminine (*n* = 6). (**D**) Quantification of relative RNF146 messenger RNA levels normalized to that of GAPDH in SH-SY5Y cells treated for 37 hrs with 10 uM of the following RNF146-inducing compounds: rhododendrin, liquiritigenin, chlorogenic acid, and piperlonguminine (*n* = 3). (**E**) Representative western blots showing RNF146 expression in SH-SY5Y cells treated for 37 hrs with the indicated compounds. β-actin serves as a loading control. (**F**) Quantification of relative RNF146 protein levels normalized to that of β-actin in SH-SY5Y cells treated for 37 hrs with 10 uM of the following RNF146-inducing compounds: rhododendrin, liquiritigenin, chlorogenic acid, and piperlonguminine (*n* = 3). Data are expressed as mean ± SEM. ^**^P < 0.01 and ^***^P < 0.001, ANOVA test followed by Tukey post hoc analysis.

A high-throughput luciferase-based screen using pGL3-RNF146-Luc was designed to identify natural compounds that activate the RNF146 promoter (Figure 3A). SH-SY5Y cells were transfected with pGL3-RNF146-Luc and pRL-TK and then plated onto 96-well plates for natural compound treatments. For this high-throughput screen, a pure single chemical compound library extracted from traditional herb medicines was used. The purity and identity of each chemical compound in the library were validated by nuclear magnetic resonance spectroscopy (NMR) and high-performance liquid chromatography (HPLC) at the National Development Institute of Korean Medicine (NDIKM). DMSO treatment and low-dose H_2_O_2_ treatment were used as negative and positive RNF146 induction controls, respectively (Figure 3A). Z’ factor values for each plate revealed that our high-throughput experiments were robust and appropriate for high-content quantitative screening (Supplementary Figure 3A). A total of 640 natural compounds were tested in the screen. For the screen, cells were treated for 37 hrs, and the fold increases in luciferase activity compared to the DMSO controls were calculated (Figure 3B). For the top four compounds with the highest induction of RNF146 promoter activity in the initial screen, the luciferase assay was repeated to validate these results (Figure 3C). These compounds were rhododendrin, liquiritigenin, chlorogenic acid, and piperlonguminine (Figure 3C and Supplementary Figure 3B). These compounds also increased RNF146 mRNA and protein levels in SH-SY5Y cells, as determined by RT-qPCR and western blotting, respectively (Figure 3D–3F). Of these four RNF146 inducers, liquiritigenin showed the most consistent ability to induce RNF146 promoter activity, mRNA, and protein expression. Therefore, we chose liquiritigenin (Supplementary Figure 4A–4C) for subsequent detailed characterization as a neuroprotective agent.

### Liquiritigenin induces RNF146 expression via estrogen receptor beta activation

**Figure 4:**
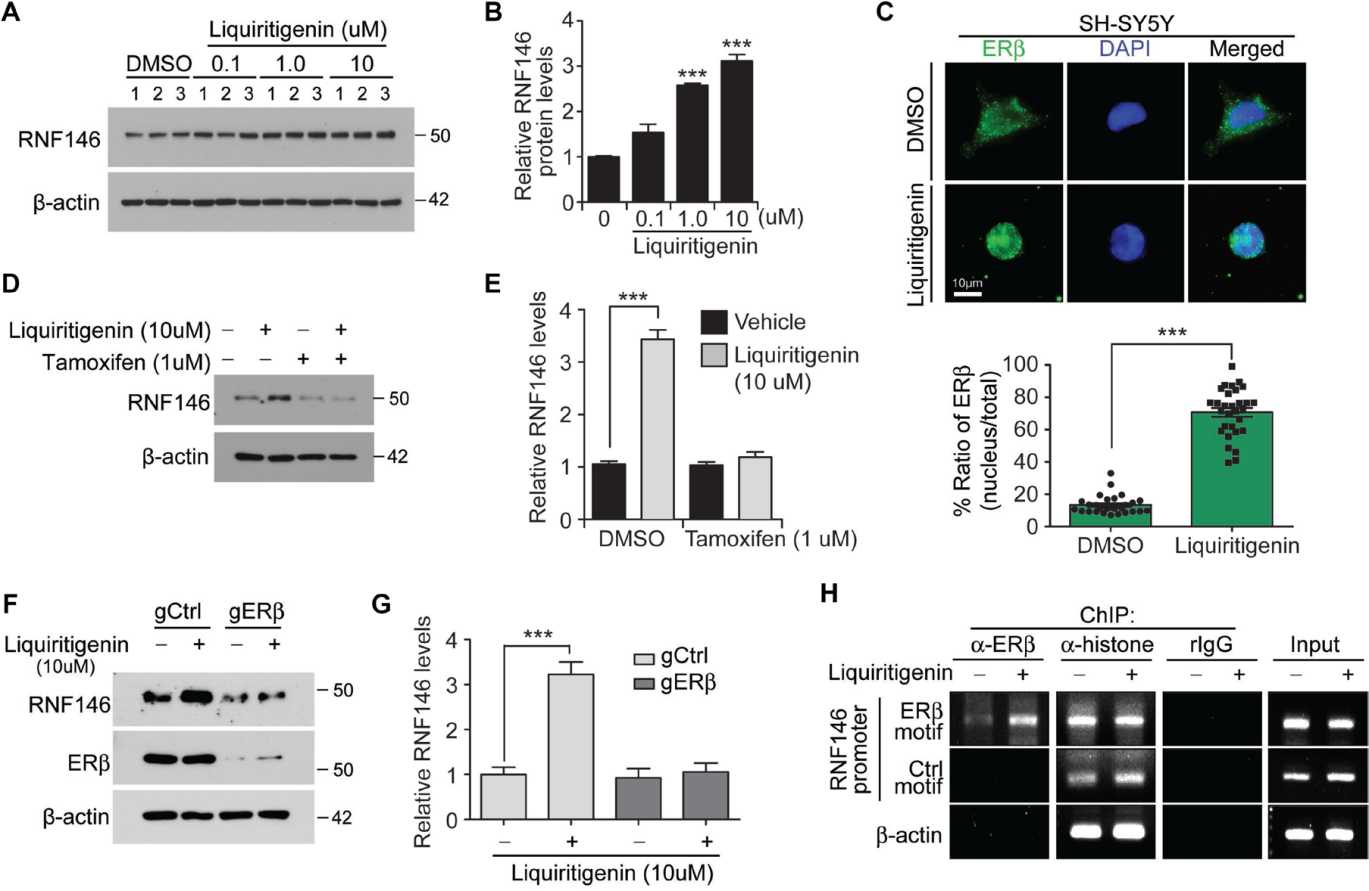
Liquiritigenin induces RNF146 expression via ER activation (**A**) Immunoblot analysis of RNF146 levels in SH-SY5Y cells treated with the indicated concentrations of liquiritigenin for 48 hrs. (**B**) Quantification of relative RNF146 expression levels (normalized to those of β-actin) from panel A (*n* = 3). (**C**) Representative immunofluorescence images showing nuclear translocation of estrogen receptor beta (ERβ) in response to liquiritigenin treatment (37 hrs) in SH-SY5Y cells. Relative distribution of ERβ in the nucleus as normalized to ERβ in the total cell area is shown in the bottom panel (*n* = 30 cells from two independent experiments). (**D**) Representative western blots showing that liquiritigenin (10 uM, 48 hrs)-mediated induction of RNF146 expression is blocked by the estrogen receptor antagonist tamoxifen (1 uM, 8 hrs pretreatment). (**E**) Quantification of relative RNF146 expression levels (normalized to those of β-actin) in the experimental groups in panel C (n = 3). (**F**) Representative western blots showing that liquiritigenin (10 uM, 48 hrs)-mediated induction of RNF146 expression is blocked by deletion of ERβ by CRISPR-cas9. (**G**) Quantification of relative RNF146 expression levels (normalized to those of β-actin) in the experimental groups in panel C (*n* = 3). (**H**) Chromatin anti-ERβ immunoprecipitation (ChIP) of putative ER responsive element (ERβ motif) within RNF146 promoter region determined by PCR using specific primers. Non ER responsive element within RNF146 promoter (Ctrl motif) and β-actin region were used as negative controls. Immunoprecipitation using either anti-histone antibodies or rabbit IgG was included as ChIP experimental controls. Data are expressed as mean ± SEM. ^*^P < 0.05, ^**^P < 0.01, and ^***^P < 0.001, unpaired two-tailed student *t* test or ANOVA test followed by Tukey post hoc analysis.

Liquiritigenin, which is found in the root of *Glycyrrhizae uralensis*, possesses estrogenic activity and exhibits neuroprotective activity against glutamate excitotoxicity [19]. Liquiritigenin increased both RNF146 mRNA and protein expression levels in a dose-dependent manner, reaching approximately three fold induction with 10 uM liquiritigenin treatment (Figure 4A, 4B and Supplementary Figure 4D). No further increase of RNF146 induction was observed in SH-SY5Y cells with higher concentrations than 10 uM liquiritigenin (Supplementray Figure 4E). To examine the molecular mechanisms by which liquiritigenin treatment induces RNF146 expression, we monitored RNF146 expression after inhibiting estrogen receptor activation. This experiment was based on the observation that liquiritigenin has been reported to be a highly selective estrogen receptor beta (ERβ) agonist [20]. Consistent with this notion, liquiritigenin treatment in SH-SY5Y cells resulted in nuclear translocation of endogenous ERβ (Figure 4C). SH-SY5Y cells were treated with liquiritigenin in the presence or absence of tamoxifen, an estrogen receptor antagonist. Tamoxifen treatment completely blocked the increase in RNF146 expression normally observed in response to liquiritigenin treatment (Figure 4D, 4E), indicating that estrogen receptor activation was responsible for liquiritigenin-mediated induction of RNF146 expression. Consistent with the western blot data was the finding that liquiritigenin-mediated induction of RNF146 mRNA expression was abolished by cotreatment with tamoxifen, as determined by RT-qPCR (Supplementary Figure 4F). The requirement of ERβ in liquiritigenin-induced RNF146 expresssion was further determined in SH-SY5Y cells by selective deletion of ERβ by CRISPR-cas9 application. Expression of both cas9 and single guided RNA (sgRNA) targeting ERβ resulted in selective ablation of ERβ without affecting ERa (Supplementary Figure 4G). In the background of ERβ deletion, liquiritigenin failed to induce RNF146 expression (Figure 4F, 4G). Interestingly, estrogen receptor activation alone seems to be sufficient for RNF146 induction because estradiol treatment increased RNF146 expression; however, persistent estradiol treatment eventually led to downregulation of RNF146 expression (Supplementary Figure 4H).

Next we examined whether ERβ indeed binds to RNF146 promoter in response to liquiritigenin treatment. Primers were designed to monitor pulling down of RNF146 promoter harboring putative ER binding motif (TGACCT) which was predicted by PROMO (Supplementary Figure 4I). Chromatin anti-ERβ immunoprecipitation (ChIP) revealed enhanced occupancy of RNF146 promoter in response to liquiritigenin treatment (Figure 4H). PCR following ChIP with anti-histone antibodies has no difference for both vehicle and liquiritigenin treatment (Figure 4H).

### Liquiritigenin-mediated induction of RNF146 expression prevents parthanatos

**Figure 5:**
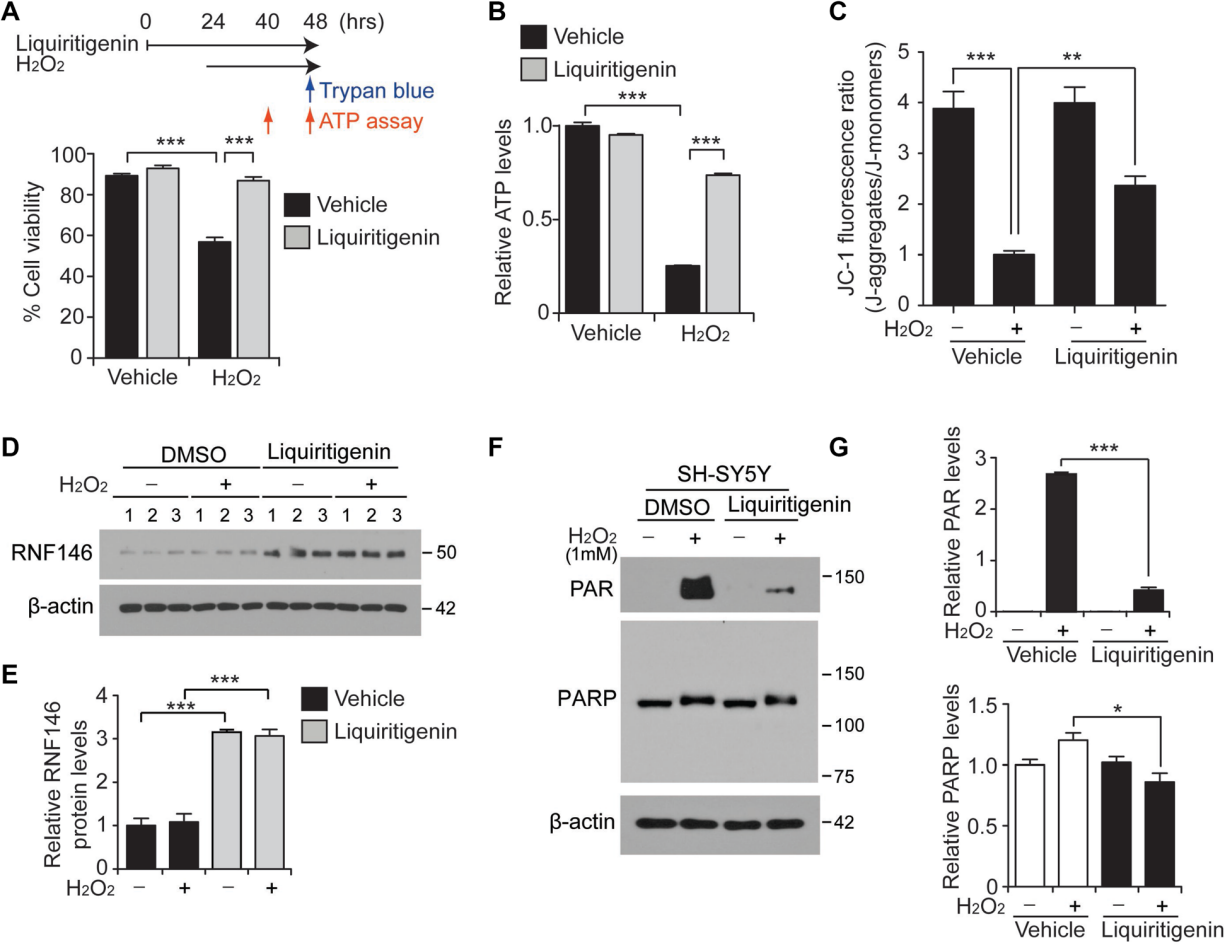
Liquiritigenin protects SH-SY5Y cells from oxidative stress via RNF146 expression and PARP1 inhibition (**A**) Trypan blue exclusion viability assay in SH-SY5Y cells demonstrating the protective effect of liquiritigenin (10 uM, 48 hrs) against H_2_O_2_ (1 mM, 24 hrs)-induced cell death (*n* = 6). The upper panel scheme summarizes the experimental design for the trypan blue cell viability assay and the ATP assessment. (**B**) Assessment of relative intracellular ATP levels in SH-SY5Y cells challenged with H_2_O_2_ (1 mM, 16 hrs) following pretreatment with liquiritigenin (10 uM, 40 hrs) or DMSO vehicle as a control (*n* = 6). (**C**) Assessment of mitochondrial membrane potential using JC-1 fluorescent dye in SH-SY5Y cells challenged with H_2_O_2_ (1 mM, 16 hrs) following pretreatment with liquiritigenin (10 uM, 37 hrs) or DMSO as a control (*n* = 7). (**D**) Representative immunoblots showing RNF146 expression levels in SH-SY5Y cells challenged with H_2_O_2_ (1mM, 24 hrs) following pretreatment with liquiritigenin (10 uM, 48 hrs) or DMSO vehicle as a control. (**E**) Quantification of relative RNF146 expression levels in the experimental groups in panel d. Values are normalized to those of β-actin (*n* = 3). (**F**) Representative immunoblots showing poly (ADP-ribose) (PAR) expression levels in SH-SY5Y cells challenged with H_2_O_2_ (1 mM, 15 min) following pretreatment with liquiritigenin (10 uM, 24 hrs) or DMSO vehicle as a control. (**G**) Quantification of relative PAR and PARP expression levels in the experimental groups in panel C. Values are normalized to those of β-actin (*n* = 3). Data are expressed as mean ± SEM. ^*^P < 0.05, ^**^P < 0.01, and ^***^P < 0.001, ANOVA test followed by Tukey post hoc analysis.

We found that RNF146 expression is cytoprotective in SH-SY5Y cells challenged with PD-related toxins. Importantly, liquiritigenin was safe and did not have any obvious cellular toxicity. Specifically, the viability of SH-SY5Y cells was not altered even upon high-dose liquiritigenin treatment (100 uM) (Supplementary Figure 5A). With this desirable therapeutic index, we next examined the molecular mechanisms of cytoprotection by liquiritigenin (an RNF146 inducer) in these models. Rather than using all toxin models for mechanism study, we chose hydrogen peroxide as a representative PD toxin since it has been widely used to model oxidative stress and DNA damage. SH-SY5Y cells were pretreated with liquiritigenin, thereby inducing RNF146 expression, and then challenged with hydrogen peroxide, which induces PARP1 activation (Figure 5A). Trypan blue cell viability assays revealed that liquiritigenin completely abolished hydrogen peroxide-induced toxicity in SH-SY5Y cells. Although this study aimed to evaluate RNF146 inducer application to PD models, it would be worthwhile to examine whether our novel RNF146 inducing compound could protect primary cortical neurons from oxidative stress. In this context, primary cultured mouse cortical neurons were exposed to liquiritigenin which led to approximate four fold increase of RNF146 expression (Supplementary Figure 5C). Consistent with cell protection in neuroblastoma cell line, liquiritigenin treatment prevented H_2_O_2_-induced neuronal toxicity (Supplementary Figure 5D, 5E).

Among the multiple mechanisms of cell death execution following PARP1 overactivation, energy depletion has been proposed to play a partial role, since sustained PARP1 activation requires energy [21]. In this context, we also monitored intracellular ATP levels at 16 hrs after hydrogen peroxide treatment. Consistent with the results of the trypan blue exclusion assay, substantial depletion of cellular ATP content was caused by hydrogen peroxide treatment; moreover, this effect was markedly reversed by liquiritigenin treatment (Figure 5B). Extended incubation of cells in the presence of hydrogen peroxide treatment (up to 24 hrs) led to almost complete depletion of intracellular ATP, whereas liquiritigenin maintained the cellular levels of ATP (Supplementary Figure 5B).

Maintenance of mitochondrial function is critical for cellular viability, and PARP1 overactivation has been shown to impair mitochondria respiration and function [22]. Thus, mitochondrial membrane potential was measured using JC-1 dye. Mitochondria potential dissipation induced by H_2_O_2_ was largely prevented by liquiritigenin treatment (Figure 5C), suggesting the role of liquiritigenin in the maintenance of mitochondria function.

Under these H_2_O_2_ stress conditions, RNF146 expression remained high upon liquiritigenin treatment, both in the presence and absence of hydrogen peroxide-mediated stress (Figure 5D, 5E). RNF146 antagonizes PARP1 activity by binding and polyubiquitinating PARsylated PARP1, thereby targeting it for proteasomal degradation [2, 6]. Hydrogen peroxide treatment for 15 minutes resulted in robust activation of PARP1, as shown by the increased PAR signal. This enhanced PAR signal was largely abolished by treatment with liquiritigenin, an RNF146 inducer (Figure 5F, 5G). PARP1 levels were also modestly reduced in this protected condition (Figure 5F, 5G). The protective effects of liquiritigenin were also evaluated upon treatment with other PD-associated toxins (6-OHDA and rotenone). These experiments showed that liquiritigenin also exerted substantial protective effects (Supplementary Figure 5F). AIMP2-induced cell toxicity was also prevented by liquiritigenin (Supplementary Figure 5G). Similar to the results from H_2_O_2_ stress paradigm, AIMP2-stimulated enhancement of PAR-conjugated proteins was substantially reduced by liquiritigenin treatment which induced RNF146 expression (Supplementary Figure 5H, 5I). Collectively, these results indicate that liquiritigenin has broad cytoprotective ability against various PARP1-activating stimuli including reactive oxygen species and AIMP2 accumulation.

### Liquiritigenin-mediated cell protection requires RNF146 and ERβ

**Figure 6:**
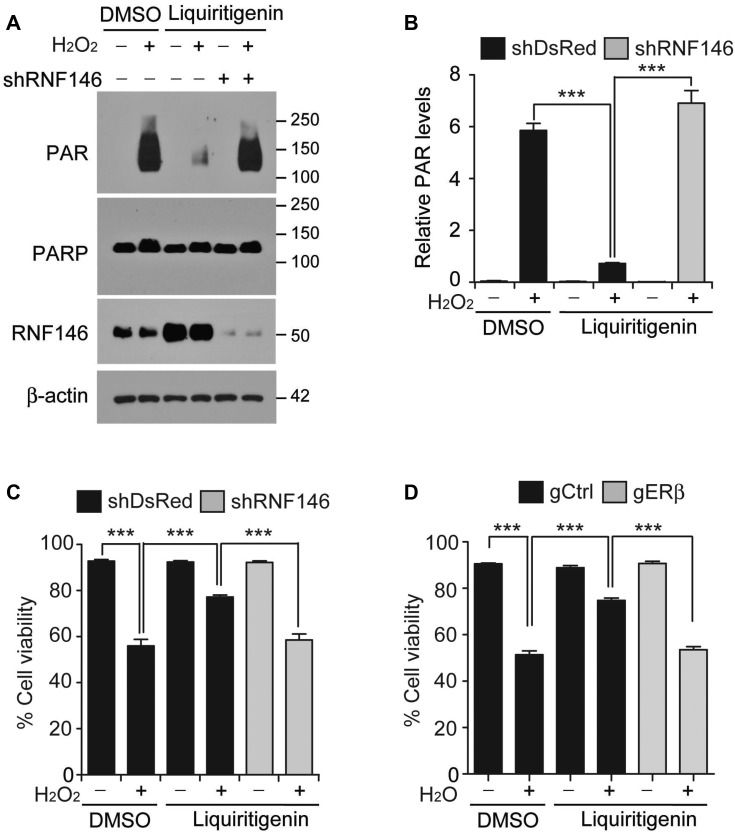
RNF146 and ERβ are required for liquiritigenin-mediated cytoprotection (**A**) Representative western blots of PAR, PARP1 and RNF146 expression levels in SH-SY5Y cells transfected with shRNA directed against RNF146 (72 hrs) and challenged with H_2_O_2_ (1 mM) for 15 min following liquiritigenin treatment (10 uM, 24 hrs). (**B**) Quantification of the relative levels of PAR expression in the experimental groups in panel A (*n* = 3). (**C**) Trypan blue exclusion viability assay in SH-SY5Y cells showing that the protective effect of liquiritigenin (10 uM, 48 hrs) against H_2_O_2_ (1 mM, 24 hrs) is abolished by shRNA-mediated knockdown of RNF146 (72 hrs) (*n* = 6). (**D**) Trypan blue exclusion viability assay in SH-SY5Y cells showing that the protective effect of liquiritigenin (10 uM, 48 hrs) against H_2_O_2_ (1 mM, 24 hrs) is abolished by CRISPR-cas9-mediated deletion of ERβ (72 hrs) (*n* = 10). Data are expressed as mean ± SEM. ^*^P < 0.05, ^**^P < 0.01, and ^***^P < 0.001, ANOVA test followed by Tukey post hoc analysis.

As a natural isoflavone compound and an agonist of estrogenic receptor beta [20], liquiritigenin may interfere with complex transcriptional programs to confer protection against PD-associated stimuli. To determine the role of RNF146 (a neuroprotective gene) induction in liquiritigenin-mediated PARP1 inhibition and cytoprotection, we silenced RNF146 expression by transfection of shRNA targeting RNF146 (shRNA-RNF146). shRNA-RNF146 expression knocked down the level of endogenous RNF146 by more than 80% (Supplementary Figure 6A). shRNA-mediated inhibition of liquiritigenin-mediated RNF146 induction resulted in a sustained increase of PAR activity following oxidative stress (Figure 6A, 6B). Cell viability was also assessed after shRNA-mediated suppression of liquiritigenin-mediated RNF146 induction to ascertain the role of RNF146 induction in the cytoprotective activity of liquiritigenin. Liquiritigenin-mediated protection of SH-SY5Y cells against hydrogen peroxide was abolished by shRNA-mediated silencing of RNF146 (Figure 6C). Importantly, the viability of liquiritigenin-treated SH-SY5Y cells exposed to hydrogen peroxide under RNF146 knockdown background was comparable to that of hydrogen peroxide-treated SH-SY5Y cells exposed to vehicle (Figure 6C). Consistent with this result, prevention of liquiritigenin-mediated intracellular ATP depletion was abolished by shRNA-mediated knockdown of RNF146 (Supplementary Figure 6B). The role of ERβ in cell viability was also assessed. Similar to RNF146 knockdown condition, CRISPR-cas9-mediated ablation of ERβ abolished liquiritigenin-induced cytoprotection against oxidative stress (Figure 6D). Together, these results confirm that liquiritigenin-mediated ERβ activation and RNF146 expression inhibits PARP1 and confers protection against PD-associated toxic insult.

### Liquiritigenin prevents DA neurodegeneration in a 6-OHDA PD model

**Figure 7:**
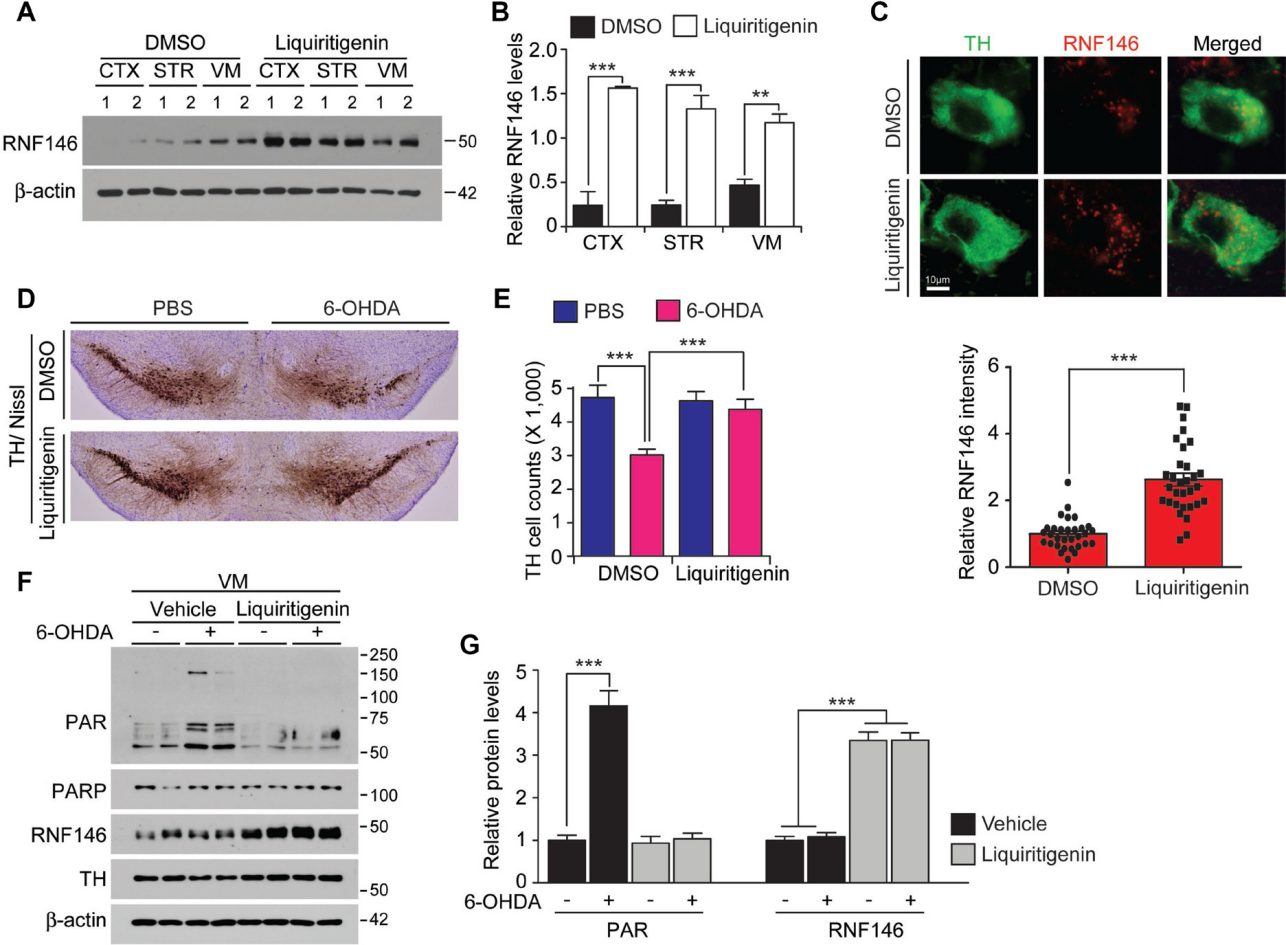
The RNF146 inducer liquiritigenin prevents dopamine neuron loss in a PD mouse model (**A**) Western blot analysis of brain regional expression of RNF146 in 2-month-old mice treated with liquiritigenin (i.p. 10 mg/kg/day, 3 days) or DMSO vehicle. Cortex, CTX; STR, striatum; VM, ventral midbrain. (**B**) Quantification of RNF146 protein expression levels in tissue samples from the indicated brain regions from 2-month-old mice treated with liquiritigenin or DMSO for three days. Values are normalized to those of β-actin (*n* = 4 mice per group). (**C**) Representative confocal immunofluorescence images of TH and RNF146 expression using the indicated antibodies in the ventral midbrain sections from 2-month-old mice treated with liquiritigenin or DMSO for three days. Bottom panel shows relative expression levels of RNF146 in TH positive dopaminergic neurons in liquiritigenin treated group as normalized to DMSO group (*n* = 31 cells per each group from three mice). (**D**) Representative tyrosine hydroxylase (TH) immunohistochemical staining of the substantia nigra of 6-OHDA PD mice treated with liquiritigenin or DMSO. 6-OHDA (8 µg) was stereotaxically injected into the striatum (coordinates from bregma, L: -2.0, AP: 0.5, DV: -3.0 mm) to model dopaminergic neurodegeneration. (**E**) Stereological assessment of tyrosine hydroxylase (TH)-positive dopaminergic neurons in the substantia nigra pars compacta of the injection side from the indicated mouse groups (*n* = 6 mice per group). (**F**) Western blot analysis of ventral midbrain protein expression of PAR, PARP1, RNF146, and TH in 2-month-old mice treated with liquiritigenin (i.p. 10 mg/kg/day, 3 days) or DMSO vehicle followed by intrastriatal 6-OHDA injection (37 hrs). (**G**) Quantification of PAR, and RNF146 protein expression levels in tissue samples from the indicated experimental groups in panel F. Values are normalized to those of β-actin (*n* = 4 mice per group). Data are expressed as mean ± SEM. ^*^P < 0.05, ^**^P < 0.01, and ^***^P < 0.001, unpaired two-tailed student *t* test or ANOVA test followed by Tukey post hoc analysis.

Liquiritigenin has been shown to penetrate into the brain for its neuroprotective function [19]. To determine whether liquiritigenin increases RNF146 expression *in vivo*, we administered liquiritigenin intraperitoneally to mice for three consecutive days and then examined RNF146 expression levels in mouse brains. The ventral midbrain demonstrated slightly higher basal expression of RNF146 mRNA and protein compared to cortex and the striatum (Figure 7A, 7B and Supplementary Figure 7). Liquiritigenin treatment resulted in substantially increased levels of RNF146 mRNA and protein expression in selected brain subregions (striatum, ventral midbrain, and cerebellum) compared to the corresponding subregions of vehicle-treated control mice (Figure 7A, 7B and Supplementary Figure 7). Importantly, liquiritigenin increased RNF146 expression in ventral midbrain dopaminergic neurons as revealed by immunofluorescence using anti-RNF146 and anti-TH antibodies (Figure 7C).

Next, we assessed the neuroprotective effects of liquiritigenin in a 6-OHDA-induced PD mouse model. Injection of 6-OHDA into the striatum is commonly used to model PD in mice and results in retrograde and selective degeneration of nigrostriatal neurons over a period ranging from several days to weeks. We found that intrastriatal injection of 6-OHDA resulted in an approximately 50 % loss of tyrosine hydroxylase (TH)-positive dopaminergic neurons in the substantia nigra pars compacta (Figure 7D, 7E). Liquiritigenin pretreatment of mice resulted in markedly enhanced dopaminergic neuronal survival after 6-OHDA intoxication (Figure 7D, 7E). Molecular alterations were also monitored by western blots to show nearly complete inhibition of PARP1 activity by liquiritigenin in 6-OHDA injection PD model (Figure 7F, 7G).

### RNF146 downregulation and PARP1 activation in PD pathogenesis

**Figure 8:**
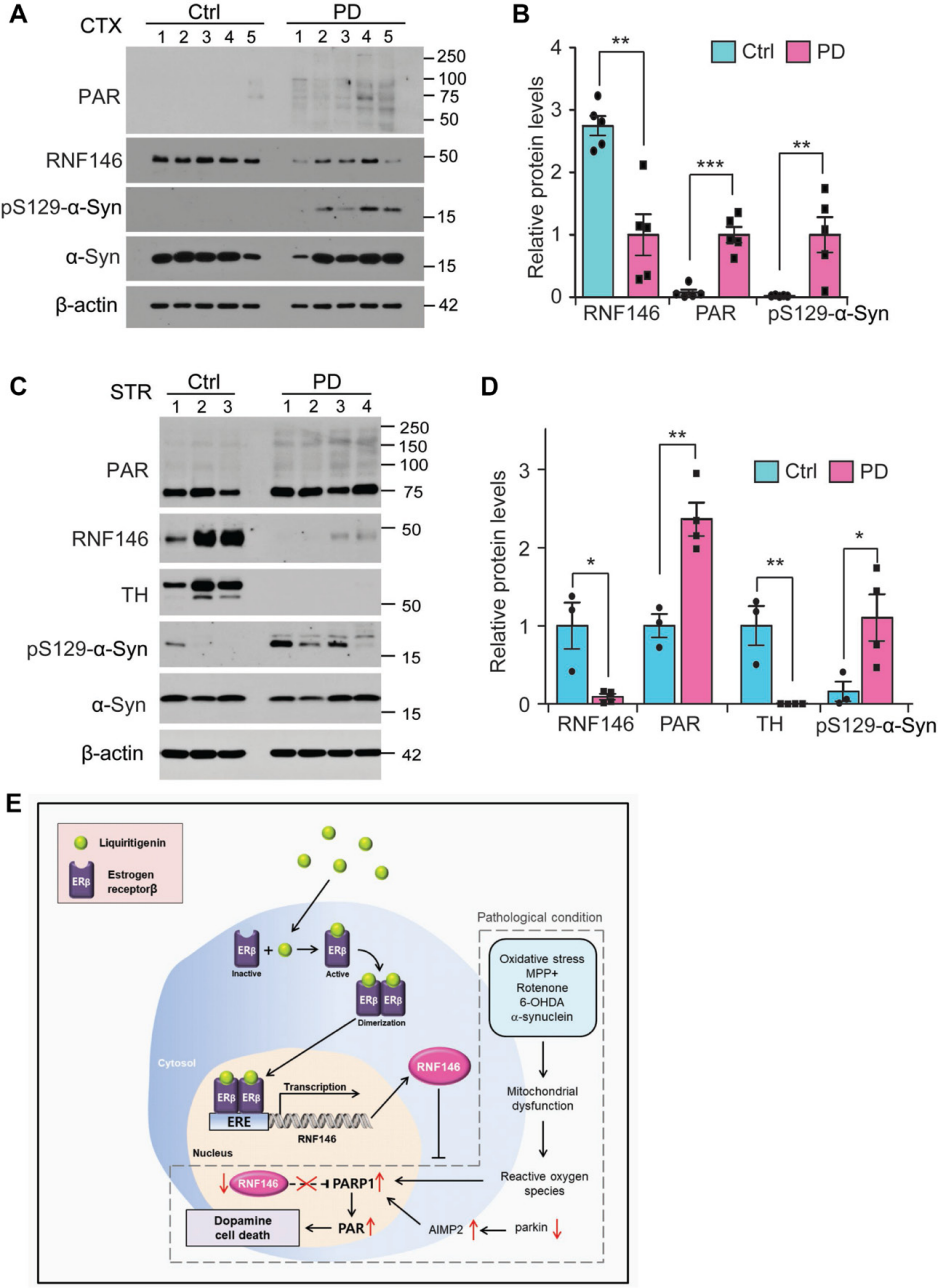
Dysregulation of RNF146-PARP1 pathways in PD pathogenesis (**A**) Expression of PARsylated proteins, RNF146 and phosphorylated α-synuclein (pS129-a-syn) in postmortem cortex brains from PD patients and age-matched control subjects (Ctrl) determined by western blots using the designated antibodies. (**B**) Quantification of relative protein expression levels normalized to β-actin in postmortem brain tissue samples (*n* = 5 per group). (**C**) Expression of PARsylated proteins, RNF146, TH and phosphorylated α-synuclein (pS129-a-syn) in postmortem striatum from PD patients and age-matched control subjects (Ctrl) determined by western blots using the designated antibodies. (**D**) Quantification of relative protein expression levels normalized to β-actin in postmortem striatum tissue samples (*n* = 3 Ctrl and 4 PD). Data are expressed as mean ± SEM. ^*^P < 0.05, ^**^P < 0.01, and ^***^P < 0.001, unpaired two-tailed student *t* test or ANOVA test followed by Tukey post hoc analysis. (**E**) Schematic summary of signaling pathway by which liquirtigenin induces RNF146 expression and confers dopaminergic neuroprotection. In PD conditions, various toxic insults contribute to progressive loss of dopamine neurons. For instance, reactive oxygen species can cause DNA damage leading to PARP1 activation when mitochondrial function is compromised by MPP^+^, rotenone, 6-OHDA, or α-synuclein fibril. Accumulation of parkin substrate AIMP2 also contributes to PARP1 overactivation. Besides, RNF146 is downregulated in PD by unknown mechanisms. These alterations are centered to exacerbate PARP1 activation and dopamine cell loss. Liquiritigenin acts by binding to estrogen receptor β which translocates into the nucleus and stimulates transcription of RNF146. RNF146 expression antagonizes PARP1 activation, thereby preventing dopamine cell loss in PD.

To determine clinical relevance of RNF146 in PD pathogenesis, we monitored its expression in postmortem brains from PD patients with dementia (Table 1). Western blot analysis revealed that RNF146 was markedly downregulated in the cortex of PD postmortem brains compared to age-matched control subjects (Figure 8A, 8B). There was elevation of PAR modified proteins in PD postmortem brains (Figure 8A, 8B), indicating enhanced PARP1 activity during pathology. Phosphorylated α-synuclein was prominent in PD patients brains, confirming Lewy pathology spreading to this brain regions. Next we sought to expand this observation in the striatum tissues (Table 2) where pathogenic axonal demise and Lewy neurites occur in PD. There was marked reduction in RNF146 expression in PD striatum with more than two fold increase of PAR-conjugated proteins (Figure 8C, 8D). The almost complete loss of TH and manifestation of pS129-α-synuclein confirmed PD pathologies in the patients postmortem tissues (Figure 8E, 8D).

**Table 1:** Information for human postmortem cortical brain samples from Brain and Body Donation Program (BBDP, used in Figure 8A, 8B)

#	Case ID	gender	Age	PMI	Pathology
1	03-11	Female	95	2.66	Control
2	13-49	Female	75	2.5	Control
3	10-63	Male	79	3	Control
4	99-02	Female	70	2	Control
5	99-44	Male	69	2.16	Control
1	02-15	Female	78	3.5	PD, dementia
2	06-44	Male	79	2.5	PD, dementia
3	07-66	Male	75	2.25	PD, dementia
4	12-42	Male	69	3.37	PD, dementia
5	13-52	Female	80	3.47	PD, cognitive impairment

PMI (hrs), post-mortem interval

**Table 2:** Information for human postmortem striatum brain samples (used in Figure 8C, 8D)

#	BRC#	gender	Age	PMI	Pathology
1	2209	Male	71	16	Control
2	2581	Female	56	7	Control
3	2590	Male	77	10	Control
1	2661	Male	73	5.5	PD w/ dementia
2	2670	Female	60	11	PD w/ dementia, AD possible
3	2680	Female	81	11	PD w/ dementia, AD possible
4	2692	Female	75	NA	PD, PART

## DISCUSSION

### Therapeutic potential of RNF146 expression in PD

This is the first report to present evidence that RNF146 induction is potentially protective against PARP1-dependent cell death stimulated by various PD-associated toxins and gene expression. As a PAR-dependent E3 ubiquitin ligase, RNF146 is involved in the beta-catenin signaling pathway in cancer and PARP1 downstream signaling in neuronal death. Although many potential RNF146 substrates have been described, thereby providing some information on the complex biological pathways in which RNF146 may be involved, self-PARsylated PARP1 appears to be the key RNF146 substrate for mediating and amplifying parthanatos, a cell death pathway. PARP1 overactivation requires energy; therefore, ATP depletion is considered to be a mechanism of parthanatic cell death [21]. However, complex signaling pathways involving apoptosis inducing factor (AIF) nuclear translocation and DNase activation have been shown to regulate cell death execution downstream of PARP1 activation [10]. Indeed, PARP1 activation has been reported in stroke, and excess stimulation-induced excitotoxicity in cortical and striatal neurons. Moreover, PARP1 inhibition provides substantial neuroprotection in these conditions [13]. In addition to influencing acute cortical and striatal neuronal death, PARP1 activation also contributes to dopaminergic neuronal death in various PD animal models, including AIMP2 transgenic mice and MPTP mice [11, 13]. Although PARP1 inhibitors can serve as potential neuroprotective agents by halting parthanatic cell loss in PD, PARP1 inhibitors also pose potentially harmful side effects since basal PARP1 activity regulates gene transcription and genome repair. In this respect, alternative strategies for selectively targeting only overactivated PARP1 could be safer and more efficient ways of controlling PARP1-dependent neurodegeneration in PD. RNF146 is a PAR-directed E3 ubiquitin ligase that recognizes and degrades activated PARP1 more efficiently than inactive PARP1 [2]; therefore, controlling RNF146 expression is a potential therapeutic strategy for treating PD. In support of this idea, RNF146 expression provided extensive cell protection against diverse PD toxins and also against expression of AIMP2, which activates PARP1 in PD (Figure 8E). In addition to PARP1 regulation by RNF146, it has been reported that RNF146 regulates cell survival via Wnt/beta-catenin or PTEN-Akt pathway [4, 5, 16]. It would be intriguing to investigate potential involvement of these pathways in the prevention of neurodegeneration by RNF146.

### First report of RNF146 inducers

Given the strong protective function of RNF146 and the relatively low expression levels of RNF146 in brain tissue, compounds that induce RNF146 could be neuroprotective in a number of neurodegenerative disorders. RNF146 inducers have not previously been reported, partly due to the lack of information on the mechanism by which the RNF146 promoter is regulated. Here we subcloned the 1.9 kb RNF146 promoter into a luciferase construct and performed a screen for RNF146-inducing natural compounds. Since the library contained pure compounds from clinically prescribed herbal medicines, compounds with the desired properties could be used for disease-oriented translational research. We identified several compounds capable of inducing RNF146 expression with cytoprotective activity. Of the compounds identified, liquiritigenin and chlorogenic acid have already been shown to be cytoprotective [19, 23–25]. Piperlonguminine is a natural alkaloid that stimulates autophagy and exhibits tumor suppression activity [26]. Rhododendrin exerts anti-inflammatory effects *in vivo* and suppresses nitric oxide production [25]. Although the present work focused on liquiritigenin as an RNF146 inducer and neuroprotective agent, further studies characterizing other inducers could shed further light on RNF146 induction mechanisms and their therapeutic potential. Piperlonguminine is of particular interest because this compound is brain-permeable and neuroprotective in rotenone and lipopolysaccharide animal models [27]. Its property to stimulate autophagy would be additional therapeutic advantege especially for neurodegenerative disease with abnormal protein aggregation. Of note, our assessment of mRNA and protein expression of RNF146 by piperlonguminine indicates potential regulation of RNF146 protein stability. Although there was a mild increase of mRNA transcription, RNF146 protein expression by piperlonguminine was almost comparable to those by liquiritigenin. Considering robust loss of RNF146 expression in PD postmortem brains, some compounds that stabilize RNF146 protein would have potential therapeutic values.

### ER activation and RNF146 expression

RNF146 expression is increased during preconditioning with low-dose H_2_O_2_, NMDA, or sublethal short duration stroke [6, 15]. It is not yet clear how the RNF146 promoter is regulated during gene transcription reprogramming. Our unbiased RNF146 inducer screen found that the estrogen receptor signaling pathway was sufficient to induce RNF146 expression. Interestingly, estrogen receptors are expressed by dopaminergic neurons and are also involved in maintaining their functions [28]. There is also a report that estradiol exerts potent disaggregase activity for α-synuclein fibril *in vitro* [29]. In this respect, modulation of the estrogen pathway and RNF146 induction by liquiritigenin or other ER activators could be employed to enhance dopaminergic cell survival under pathological conditions. However, it is not clear whether preconditioning-induced RNF146 induction is also regulated by the ER pathway. Further research is required to fully understand RNF146 transcriptional regulation.

### RNF146 deficits in PD pathogenesis

PARP1 overactivation has been seen in the ventral midbrain of postmortem PD patients brain [11]. However, this is the first report to observe evidence of PARP1 activation in the cortex regions from postmortem PD (with dementia) patients brains. It has been noted that α-synuclein pathology propagates from brain stem to the cortical regions as the disease progresses. As such at the end stage of PD, wide spread α-synuclein pathology is present in various brain regions, causing nonmotor neurological impairments including dementia [30–33]. It is likely that α-synuclein aggregation in cortical regions of PD patients brain contributed to PARP1 overactivation. Although molecular mechanism underlying this consequence remains to be elucidated, mitochondrial dysfunction and oxidative stress by α-synuclein aggregation might have contributed to PARP1 activation [34]. Notably, there was downregulation of PARP1 antagonist RNF146 expression in PD brains, indicating clinical relevance of RNF146 deficit in PD pathogenesis. Inadequate activity of RNF146 might further exacerbate PARP1 activation and neuronal toxicity in PD.

### Liquiritigenin as a potential therapeutic for PD treatment

Liquiritigenin and its analogs have been studied for their therapeutic potential in animal models of several diseases [35]. Interestingly, liquiritigenin is brain-permeable and has been shown to be protective against cell toxicity induced by various stimuli [36, 37]. However, it is unknown whether liquiritigenin-mediated estrogen receptor activation and induction of RNF146 expression are cytoprotective in other pathological contexts. Nevertheless, our results suggest that liquiritigenin could be applied to treat neurodegenerative diseases including PD, although protective efficacy of liquiritigenin should be investigated in various PD-related cell and animal models.

ER activation is associated with a potential risk for tumor growth [20, 38]; therefore, caution must be taken when using ER agonists to treat neurodegenerative disease. Notably, liquiritigenin has been shown to selectively activate ER beta and not ER alpha [20]. This selective ER pathway activation by liquiritigenin avoids aberrant tumor growth *in vivo* [20], thereby yielding an improved safety profile of long-term liquiritigenin treatment of neurodegenerative disorders. Supporting safe clinical application of liquiritigenin for neurodegenerative diseases, several studies showed that liquiritigenin inhibited tumor growth and induced cancer cell death [39, 40]. Development of ER beta receptor agonists or liquiritigenin derivatives could potentially lead to the development of more potent and safer medications for the treatment of PD.

## MATERIALS AND METHODS

### Chemicals and antibodies

The natural compound library (640 natural compounds with purity higher than 80%, 1 mg/ml concentration) was provided from the National Development Institute of Korean Medicine (NIKOM). This library was used in the RNF146 inducer high-throughput screen. Natural compounds shown to induce RNF146 (liquiritigenin, rhododendrin, and piperlonguminine) were extracted from the appropriate herbal medicine, purified, and HPLC validated by NIKOM. Chlorogenic acid, H_2_O_2_, 6-OHDA, rotenone, and the PARP inhibitor 3AB were purchased from Sigma. Tamoxifen was purchased from Selleck Chemicals.

The following primary antibodies were used: mouse antibody to RNF146 (N201/35, 1:5000, NeuroMab), rabbit antibody to GFP (cat# 2956, 1:5000, Cell Signaling Technology), mouse antibody to PAR (cat# 4335-MC-100, 1:3000, Trevigen), rabbit antibody to PARP1 (cat# 9542S, 1:3000, Cell Signaling), rabbit antibody to estrogen receptor beta (cat#PA1-311, 1:3000, Invitrogen), rabbit antibody to estrogen receptor alpha (cat#sc-7207, 1:3000, Santa Cruz), rabbit antibody to tyrosine hydroxylase (NB300-109, 1:2000, Novus Biologicals), mouse antibody to α-synuclein (1:3,000, BD transduction), and mouse antibody to S129-phosphorylated α-synuclein (pSyn#64, 1:3,000, Wako laboratory Chemicals). For secondary antibodies, we used horse radish peroxidase (HRP)-conjugated sheep antibody to mouse IgG (cat# RPN4301, 1:5000, GE Healthcare), HRP-conjugated donkey antibody to rabbit IgG (cat# RPN4101, 1:5000, GE Healthcare), biotin-conjugated goat antibody to rabbit IgG (cat# BA-1000, 1:1000, Vector Laboratories), and HRP-conjugated mouse antibody to β-actin (cat# A3854, 1:10000, Sigma-Aldrich)

### Purification of liquiritigenin from *Glycyrrhiza uralensis*

The root of *Glycyrrhiza uralensis* was purchased from Human herb in Gyeongsan, Gyeongbuk, Korea, in July 2013. Dried roots of *Glycyrrhiza uralensis* L. (250 g) were extracted with 95% EtOH for 3 hr (500 ml), and extraction was repeated twice. The EtOH extract (42 g) was then suspended in H_2_O (500 mL) and solvent partitioned with equal volume of dichloromethane (CH_2_Cl_2_). The CH_2_Cl_2_ soluble fraction (8.9 g) was loaded on a silica gel column (70–230 mesh, 3.8×50 cm) in *n*-hexane:EtOAc = 30:1 mixture, and purified with stepwise gradient mobile phase solvent (*n*-Hexane:EtOAc = 30:1∼1:1) to produce 5 fractions. The active compound, liquiritigenin (140 mg, 0.056%) was obtained from fraction 4 and identified by comparing its various spectroscopic techniques, including UV absorption (Supplementary Figure 4A), ^1^H and ^13^C NMR spectrum (Supplementary Figure 4B, 4C) with the before literature [20]. The purity of liquiritigenin was determined as 99.0% by HPLC-ELSD system (Supplementary Figure 4A).

### HPLC-ELSD and NMR of liquiritigenin

High performance liquid chromatography (HPLC) was performed on an Agilent 1260 series (Agilent Technologies, USA) with evaporative light scattering detector (ELSD) and Kinetex C_18_ column (4.6 × 150 mm, Phenomenex), and the flow rate was set at 0.5 mL/min. The mobile phase used for the separation consisted of solvent A (0.1% trifluoroacetic acid in water) and solvent B (0.1% trifluoroacetic acid in acetonitrile). A gradient elution procedure was used as 0 min 2% B, 3 min 5% B, and 30 min 100% B. The injection volume was 3 μL for analysis. The ^1^H and ^13^C NMR spectra were recorded on a Jeol ECA-500 MHz NMR instrument, operating at 500 MHz for ^1^H NMR and 125 MHz for ^13^C NMR (Jeol, Tokyo, Japan) with tetramethylsilane as internal standard. High performance liquid chromatography (HPLC) was performed on a Agilent 1260 series (Agilent Technologies, USA) with evaporative light scattering detector(ELSD). Liquiritigenin : colorless crystals; EI-MS *m*/*z* = 256.25 [M]^+^, molecular formula C_15_H_12_O_4_; ^1^H-NMR (500 MHz, CD_3_OD) δ : 7.71 (1H, d, *J* = 8.8 Hz, H-5), 7.31 (2H, d, *J* = 8.4 Hz, H-2’), 6.82 (2H, d, *J* = 8.4 Hz, H-3′, 5′), 6.5 (1H, dd, *J* = 8.8, 2 Hz, H-6), 6.35 (1H, d, *J* = 2 Hz, H-8), 5.36 (1H, dd, *J* = 13.2, 2.8 Hz, H-2), 3.05(1H, dd, *J* = 16.8, 13.2 Hz, H-3a), 2.26 (1H, dd, *J* =16.8, 2.8 Hz, H-3b); ^13^C-NMR (125 MHz, CD_3_OD) δ : 193.6 (C-4), 166.9 (C-7), 165.6 (C-9), 159.0 (C-4′), 131.4 (C-1’), 129.9 (C-5), 129.1 (C-2’, 6’), 116.4 (C-3′, 5′), 115.0 (C-10), 111.8 (C-6), 103.9 (C-8), 81.1 (C-2), 45.0 (C-3).

### Cell culture and transfection

Human neuroblastoma SH-SY5Y cells (ATCC, Manassas, VA) were grown in DMEM containing 10% FBS (vol/vol) and antibiotics (penicillin-streptomycin 100 U/ml, ThermoFisher Scientific). Cells were propagated in a humidified atmosphere consisting of 5% CO2/95% air and maintained at 37 °C. For transient transfections of the indicated vectors, X-tremeGENE HP transfection reagents (Roche) were used according to the manufacturer’s instructions. Primary cortical cell cultures were prepared from gestational day 15 mouse embryos as previously described [6]. Experiments were performed at DIV (day *in vitro*) 14.

### Plasmids

The RNF146 promoter luciferase reporter construct (pGL3-RNF146-Luc) was generated by subcloning the PCR-amplified RNF146 promoter (-1941 bp∼-1bp from the transcription start site; amplified from genomic DNA extracted from SH-SY5Y cells) into the pGL3 luciferase backbone (Promega). CRISPR-cas9 construct targeting human ERβ was generated by cloning sgRNA sequence for ERβ (CACCGTCTGCAGCGATTACGCATC) into lentiCRISPR-v2 plasmid (Addgene plasmid #52961). The construct integrity was validated by sequencing. pEGFP-C3-AIMP2 [11], pLKO-shRNA targeting RNF146, pLKO-shRNA targeting dsRed, pEGFP-C2-human RNF146, and pEGFP-C2 [6] have been previously described.

### Western blotting

Total protein lysates were prepared by adding lysis buffer (1% Nonidet P40 in phosphate-buffered saline (PBS), pH 7.4) supplemented with protease/phosphatase inhibitors to SH-SY5Y cells washed briefly with ice-cold PBS. After three freeze and thaw cycles in dry ice, samples were centrifuged at 14,000 g for 30 min. Next, the supernatants were mixed with 2X Laemmli buffer (Bio-Rad) supplemented with b-mercaptoethanol (Sigma). After boiling the samples for 5 minutes, proteins were separated by SDS-PAGE and transferred to nitrocellulose membranes for immunoblotting. The blotted nitrocellulose membranes were stained with Ponceau (Sigma) to verify uniform protein transfer. Immunoblotting was performed with the designated antibodies, and immunoreactive bands were visualized via chemiluminescence (Pierce). Densitometric analyses of the bands were performed using ImageJ (NIH, http://rsb.info.nih.gov/ij/).

### Luciferase assay

SH-SY5Y cells were transiently cotransfected with pGL3-RNF146-Luc and pRL-TK (Promega). Cells were harvested at 37 hrs following treatments with each compound, and lysates were assayed for firefly luciferase activity using the Dual Luciferase Reporter Assay System (Promega, Madison, WI) with a microplate luminometer (Berthold Technologies) according to the manufacturer’s instructions. Firefly luciferase levels were normalized to those of the *Renilla* control. As a negative control, cells were treated with 0.1% DMSO. Luciferase values for each chemical treatment were normalized to that of the DMSO control.

### High-throughput luciferase screening

At 24 hrs after pGL3-RNF146-Luc and pRL-TK transfection, SH-SY5Y cells were plated onto 96-well white, flat-bottom plates. Cells were seeded at 80% confluency in 100 µl of DMEM (10 % FBS plus penicillin/streptomycin) and incubated at 37°C in a 5% CO_2_ atmosphere. The next day, each compound (640 natural compounds purified from herbal medicines, NDIKM) was added to 50 µl pre-warmed DMEM at a final concentration of 20 mM. Half of the medium was replaced with pre-warmed DMEM containing the test compound (final compound concentration, 10 mM). After 37 hrs of incubation, luciferase activity was measured using the Dual Luciferase Reporter Assay System (Promega, Madison, WI). Each plate had multiple wells of positive controls (100 uM H_2_O_2_, 10 min preconditioning with recovery for 24 hrs) and negative controls. To assess the high-throughput screening readiness and robustness of our assay, the Z’ factor was quantified as follows: Z’ factor = 1-3(SDp+SDn)/(MEANp-MEANn), where MEANp and MEANn are the mean values of the positive control (H_2_O_2_ preconditioning) and the negative control (no preconditioning) in the assay, and SDp and SDn are the standard deviations of the positive and negative controls, respectively [41, 42]. An HTS-ready assay should have a Z′ factor between 0.5 and 1.

### Real-time quantitative PCR

Total RNA was extracted with QIAzol Lysis Reagent (cat# 79306, QIAGEN) and then treated with DNase I to eliminate trace DNA contamination. cDNA was synthesized from total RNA (1.5 ug) using a first-strand cDNA synthesis kit (iScript cDNA synthesis kit, Bio-Rad). The relative quantities of mRNA expression were analyzed using real-time PCR (QuantStudio 6 flex Real-Time PCR System, Applied Biosystems). SYBR Green PCR master mix (Cat# 4309155, Applied Biosystems) was used according to the manufacturer’s instructions. The relative mRNA expression levels of target genes were calculated by the DDCt method [43] using GAPDH as an internal loading control. The primer sequences for real-time gene amplification are as follows:

*hGAPDH*: F- AAACCCATCACCATCTTCCAG, R- AGGGGCCATCCACAGTCTTCT;
*hRNF146*: F- ATTCCCGAGGATTTCCTTGACA, R- GCTCATCGTACTGCCACCA.
*mGAPDH*: F- TGGCCTTCCGTGTTCCTAC, R- GAGTTGCTGTTGAAGTCGCA;
*mRNF146*: F- AGTCCTGTTCCAATACTGCACC, R- GAAGCACCCTTTACACACAGAT.

### Chromatin immunoprecipitation

Chromatin immunoprecipitation was carried out according to manufacturer’s instruction (Millipore) with modification. Briefly, SH-SY5Y cells (treated with DMSO or liquiritigenin) were fixed with 1% formaldehyde for 10 min at 37°C. Glycerol quenched samples were lysed in 1 ml of SDS buffer containing protease inhibitors. The lysates were incubated for 10 min on ice and sonicated to shear DNA. The samples were centrifuged at 10,000 x g at 4°C for 10 min and supernatant was taken. Pre-cleared samples were incubated with either anti-ERβ, anti-histone antibodies or rabbit IgG (rIgG)-agarose bead followed by a number of washes. Elutes were subjected to reverse cross-linking and DNA was recovered by phenol-chloroform-ethanol purification. PCR was performed using template DNA and the following primers:

Putative ERβ binding motif (TGACCT) within RNF146 promoter (F-CGAGTAGCTGGGATTACAGGC; R- ACACACTTAAAGAGGTTCTCTGTA),

RNF146 promoter-control region (F-GCGCAAGCATCACTGAACTA; R- TGTTGCATTTTGGGATTTCA),

β-actin region (F- AGAGCTACGAGCTGCCTGAC; R- AGCACTGTGTTGGCGTACAG)

### Cell viability assay

SH-SY5Y cells were plated in 6-well plates at a density of 0.5 × 10^6^ cells per well. Following transient transfection with the indicated constructs, cells were grown in DMEM containing low serum (2.5 % FBS), with or without chemicals at the indicated concentrations, for the indicated durations. Next the cells were harvested by trypsinization, thereby yielding single cell suspensions. The cells were washed twice with PBS and then resuspended in serum-free DMEM. Resuspended cells were mixed with an equal volume of 0.4% trypan blue (wt/vol) and incubated for 2 min at room temperature. Live and dead cells were counted using a Countess II Automated Cell Counter (Life Technologies). Alternatively, intracellular ATP levels were measured with a microplate luminometer (Berthold Technologies) using the CellTiter-Glo Luminescent Cell Viability Assay (Promega). Cell Counting Kit-8 (CCK8, DOJINDO Molecular Technologies) was also used to assess viability of SH-SY5Y cells or primary cultured neurons following the manufacturer’s instructions.

### Animal experiments

All animal experiments were approved by the Ethical Committee of Sungkyunkwan University and were conducted in accordance with all applicable international guidelines. Male C57BL/6N mice (2 months old) were obtained from Orient (Suwon, Korea). Animals were maintained on a 12-h dark/light cycle in air-controlled rooms. Mice were provided *ad libitum* access to diet and water. All efforts were made to minimize animal suffering and to minimize the number of animals used. Liquiritigenin was administered to mice intraperitoneally. Liquiritigenin administration (10 mg/kg body weight, i.p.) began on day 0 and was continued for 7 days, followed by stereological assessment of dopamine neuron counts. Intrastriatal injection of 6-OHDA was performed on day 3. Mice brains were prepared for analysis as described below.

### Intrastriatal injection of 6-OHDA

For stereotaxic injection of 6-hydroxy dopamine (6-OHDA, 8 µg), 2-month-old C57/BL6N mice treated either with liquiritigenin for 3 days or DMSO as a control were anesthetized with pentobarbital (60 mg/kg). The 6-OHDA injection procedure was performed as described previously [44], but with some modifications. Briefly, an injection cannula (26.5 gauge) was applied stereotaxically into the striatum (anteroposterior, 0.5 mm from bregma; mediolateral, 2.0 mm; dorsoventral, 3.0 mm) and unilaterally applied into the right hemisphere. Infusion was performed at a rate of 0.2 µl/min. A total of 2 µl of 6-OHDA (4 µg/µl in sterile PBS) was injected into each mouse. After the final injection, the injection cannula was maintained in the striatum for an additional 5 minutes for complete absorption of the chemical. The cannula was then slowly removed from the mouse brain. Head skin was closed by suturing. Wound healing and recovery were monitored following the surgery. For stereological analysis, animals were perfused at 4 days after intrastriatal 6-OHDA injection and fixed intracardially with ice-cold PBS. Next, tissue was fixed with 4% paraformaldehyde. Mouse brains were removed and processed for immunohistochemistry.

### Preparation of tissues for immunoblotting

Mice were euthanized by cervical dislocation. Mouse brain subregions (CTX, VM, STR) were located following procedures described previously [45]. The frontal cortex was dissected and designated as CTX. Mouse brain tissues were homogenized in lysis buffer [10 mM Tris-HCl, pH 7.4, 150 mM NaCl, 5 mM EDTA, 0.5% Nonidet P-40, 10 mM Na-b-glycerophosphate, Phosphate Inhibitor Cocktails I and II (Sigma), and a complete protease inhibitor mixture (Roche)] using a Diax 900 homogenizer. After homogenization, samples were rotated at 4°C for 30 min to ensure complete lysis. The homogenates were then centrifuged at 52,000 rpm for 20 min, and the resulting supernatants were collected. Protein levels were quantified using the BCA Protein Assay Kit (Pierce) with BSA standards. Proteins were then subjected to immunoblotting with the antibodies of interest. Immuno-reactive bands were visualized with an enhanced chemiluminescence kit (Pierce). Densitometric analyses of protein bands were performed using ImageJ (NIH, http://rsb.info.nih.gov/ij/).

### TH stereological cell counting

After the scheduled treatments with liquiritigenin (10 mg/kg body weight, daily intraperitoneal administration), animals of striatal injection with 6-OHDA (intoxication model) or PBS (controls) were anesthetized with pentobarbital (50 mg/kg, intraperitoneal injection) and perfused with PBS. Next, the tissue was fixed with 4% paraformaldehyde (wt/vol in PBS). Brains were post-fixed overnight with 4% paraformaldehyde and subsequently cryoprotected overnight in 30% sucrose in PBS (wt/vol). Coronal sections (thickness of 40 um) were cut through the brain including the substantia nigra. Every fourth section was used for analysis. For analysis of tyrosine hydroxylase (TH) expression, sections were incubated with a 1:1000 dilution of rabbit polyclonal anti-TH (Novus) antibody followed by sequential incubations with biotinylated goat anti-rabbit IgG and streptavidin-conjugated horseradish peroxidase (HRP) using a Vectastain ABC kit (Vector Laboratories, Burlingame, CA) according to the manufacturer’s instructions. To visualize TH-positive cells, 3,3-diaminobenzidine (DAB, cat# D4293, Sigma) was used as an HRP substrate. Immunostained brain sections were counterstained with Nissl. The total number of TH-positive neurons in the substantia nigra pars compacta was determined using the Optical Fractionator probe in Stereo Investigator software (MicroBrightfield, Williston, VT). All stereological counting was performed in a blinded manner to mouse treatments.

### Statistics

Quantitative data are presented as mean ± SEM. Power analysis was performed by using G*Power 3.1 software to determine approximate sample sizes for tyrosine hydroxylase stereological counting. On the basis of mean difference from our preliminary experiments, a total sample size of four mice was calculated to potentially obtain significant difference (effect size f = 22.42 for 45% mean difference; a = 0.05). Statistical significance was assessed either via an unpaired two-tailed Student’s *t-test* (two-group comparisons) or an ANOVA test with Tukey’s HSD post hoc analysis (comparisons of more than three groups). Differences with a *P value* < 0.05 were considered significant. GraphPad Prism software was used for preparation of all plots and all statistical analyses.

### Data availability

All relevant data are available from the authors on reasonable request.

## SUPPLEMENTARY MATERIALS FIGURES



## References

[1] DaRosa PA, Wang Z, Jiang X, Pruneda JN, Cong F, Klevit RE, Xu W (2015). Allosteric activation of the RNF146 ubiquitin ligase by a poly(ADP-ribosyl)ation signal. Nature.

[2] Kang HC, Lee YI, Shin JH, Andrabi SA, Chi Z, Gagne JP, Lee Y, Ko HS, Lee BD, Poirier GG, Dawson VL, Dawson TM (2011). Iduna is a poly(ADP-ribose) (PAR)-dependent E3 ubiquitin ligase that regulates DNA damage. Proc Natl Acad Sci U S A.

[3] Zhou ZD, Chan CH, Xiao ZC, Tan EK (2011). Ring finger protein 146/Iduna is a poly(ADP-ribose) polymer binding and PARsylation dependent E3 ubiquitin ligase. Cell Adh Migr.

[4] Li N, Zhang Y, Han X, Liang K, Wang J, Feng L, Wang W, Songyang Z, Lin C, Yang L, Yu Y, Chen J (2015). Poly-ADP ribosylation of PTEN by tankyrases promotes PTEN degradation and tumor growth. Genes Dev.

[5] Zhang Y, Liu S, Mickanin C, Feng Y, Charlat O, Michaud GA, Schirle M, Shi X, Hild M, Bauer A, Myer VE, Finan PM, Porter JA (2011). RNF146 is a poly(ADP-ribose)-directed E3 ligase that regulates axin degradation and Wnt signalling. Nat Cell Biol.

[6] Andrabi SA, Kang HC, Haince JF, Lee YI, Zhang J, Chi Z, West AB, Koehler RC, Poirier GG, Dawson TM, Dawson VL (2011). Iduna protects the brain from glutamate excitotoxicity and stroke by interfering with poly(ADP-ribose) polymer-induced cell death. Nat Med.

[7] Callow MG, Tran H, Phu L, Lau T, Lee J, Sandoval WN, Liu PS, Bheddah S, Tao J, Lill JR, Hongo JA, Davis D, Kirkpatrick DS (2011). Ubiquitin ligase RNF146 regulates tankyrase and Axin to promote Wnt signaling. PLoS One.

[8] Gibson BA, Kraus WL (2012). New insights into the molecular and cellular functions of poly(ADP-ribose) and PARPs. Nat Rev Mol Cell Biol.

[9] David KK, Andrabi SA, Dawson TM, Dawson VL (2009). Parthanatos, a messenger of death. Front Biosci (Landmark Ed).

[10] Fatokun AA, Dawson VL, Dawson TM (2014). Parthanatos: mitochondrial-linked mechanisms and therapeutic opportunities. Br J Pharmacol.

[11] Lee Y, Karuppagounder SS, Shin JH, Lee YI, Ko HS, Swing D, Jiang H, Kang SU, Lee BD, Kang HC, Kim D, Tessarollo L, Dawson VL (2013). Parthanatos mediates AIMP2-activated age-dependent dopaminergic neuronal loss. Nat Neurosci.

[12] Martire S, Mosca L, d’Erme M (2015). PARP-1 involvement in neurodegeneration: A focus on Alzheimer’s and Parkinson’s diseases. Mech Ageing Dev.

[13] Mandir AS, Przedborski S, Jackson-Lewis V, Wang ZQ, Simbulan-Rosenthal CM, Smulson ME, Hoffman BE, Guastella DB, Dawson VL, Dawson TM (1999). Poly(ADP-ribose) polymerase activation mediates 1-methyl-4-phenyl-1, 2,3,6-tetrahydropyridine (MPTP)-induced parkinsonism. Proc Natl Acad Sci U S A.

[14] Yokoyama H, Kuroiwa H, Tsukada T, Uchida H, Kato H, Araki T (2010). Poly(ADP-ribose)polymerase inhibitor can attenuate the neuronal death after 1-methyl-4-phenyl-1,2,3,6-tetrahydropyridine-induced neurotoxicity in mice. J Neurosci Res.

[15] Dai C, Liang D, Li H, Sasaki M, Dawson TM, Dawson VL (2010). Functional identification of neuroprotective molecules. PLoS One.

[16] Gao Y, Song C, Hui L, Li CY, Wang J, Tian Y, Han X, Chen Y, Tian DL, Qiu X, Wang E (2014). Overexpression of RNF146 in non-small cell lung cancer enhances proliferation and invasion of tumors through the Wnt/beta-catenin signaling pathway. PLoS One.

[17] Storch A, Kaftan A, Burkhardt K, Schwarz J (2000). 6-Hydroxydopamine toxicity towards human SH-SY5Y dopaminergic neuroblastoma cells: independent of mitochondrial energy metabolism. J Neural Transm (Vienna).

[18] Watabe M, Nakaki T (2004). Rotenone induces apoptosis via activation of bad in human dopaminergic SH-SY5Y cells. J Pharmacol Exp Ther.

[19] Yang EJ, Park GH, Song KS (2013). Neuroprotective effects of liquiritigenin isolated from licorice roots on glutamate-induced apoptosis in hippocampal neuronal cells. Neurotoxicology.

[20] Mersereau JE, Levy N, Staub RE, Baggett S, Zogovic T, Chow S, Ricke WA, Tagliaferri M, Cohen I, Bjeldanes LF, Leitman DC (2008). Liquiritigenin is a plant-derived highly selective estrogen receptor beta agonist. Mol Cell Endocrinol.

[21] Cosi C, Marien M (1999). Implication of poly (ADP-ribose) polymerase (PARP) in neurodegeneration and brain energy metabolism. Decreases in mouse brain NAD+ and ATP caused by MPTP are prevented by the PARP inhibitor benzamide. Ann N Y Acad Sci.

[22] Szczesny B, Brunyanszki A, Olah G, Mitra S, Szabo C (2014). Opposing roles of mitochondrial and nuclear PARP1 in the regulation of mitochondrial and nuclear DNA integrity: implications for the regulation of mitochondrial function. Nucleic Acids Res.

[23] Choi EM, Suh KS, Lee YS (2014). Liquiritigenin restores osteoblast damage through regulating oxidative stress and mitochondrial dysfunction. Phytother Res.

[24] Jeon YJ, Kim BH, Kim S, Oh I, Lee S, Shin J, Kim TY (2013). Rhododendrin ameliorates skin inflammation through inhibition of NF-kappaB, MAPK, and PI3K/Akt signaling. Eur J Pharmacol.

[25] Kim MH, Nugroho A, Choi J, Park JH, Park HJ (2011). Rhododendrin, an analgesic/anti-inflammatory arylbutanoid glycoside, from the leaves of Rhododendron aureum. Arch Pharm Res.

[26] Makhov P, Golovine K, Teper E, Kutikov A, Mehrazin R, Corcoran A, Tulin A, Uzzo RG, Kolenko VM (2014). Piperlongumine promotes autophagy via inhibition of Akt/mTOR signalling and mediates cancer cell death. Br J Cancer.

[27] He H, Guo WW, Xu RR, Chen XQ, Zhang N, Wu X, Wang XM (2016). Alkaloids from piper longum protect dopaminergic neurons against inflammation-mediated damage induced by intranigral injection of lipopolysaccharide. BMC Complement Altern Med.

[28] Sawada H, Ibi M, Kihara T, Urushitani M, Honda K, Nakanishi M, Akaike A, Shimohama S (2000). Mechanisms of antiapoptotic effects of estrogens in nigral dopaminergic neurons. Faseb J.

[29] Hirohata M, Ono K, Morinaga A, Ikeda T, Yamada M (2009). Anti-aggregation and fibril-destabilizing effects of sex hormones on alpha-synuclein fibrils *in vitro*. Exp Neurol.

[30] Luk KC, Song C, O’Brien P, Stieber A, Branch JR, Brunden KR, Trojanowski JQ, Lee VM (2009). Exogenous alpha-synuclein fibrils seed the formation of Lewy body-like intracellular inclusions in cultured cells. Proc Natl Acad Sci U S A.

[31] Lang AE, Lozano AM (1998). Parkinson’s disease. First of two parts. N Engl J Med.

[32] Lang AE, Lozano AM (1998). Parkinson’s disease. Second of two parts. N Engl J Med.

[33] Luk KC, Kehm V, Carroll J, Zhang B, O’Brien P, Trojanowski JQ, Lee VM (2012). Pathological alpha-synuclein transmission initiates Parkinson-like neurodegeneration in nontransgenic mice. Science.

[34] Di Maio R, Barrett PJ, Hoffman EK, Barrett CW, Zharikov A, Borah A, Hu X, McCoy J, Chu CT, Burton EA, Hastings TG, Greenamyre JT (2016). alpha-Synuclein binds to TOM20 and inhibits mitochondrial protein import in Parkinson’s disease. Sci Transl Med.

[35] Bhullar KS, Rupasinghe HP (2013). Polyphenols: multipotent therapeutic agents in neurodegenerative diseases. Oxid Med Cell Longev.

[36] Kim SC, Byun SH, Yang CH, Kim CY, Kim JW, Kim SG (2004). Cytoprotective effects of Glycyrrhizae radix extract and its active component liquiritigenin against cadmium-induced toxicity (effects on bad translocation and cytochrome c-mediated PARP cleavage). Toxicology.

[37] Kim YW, Ki SH, Lee JR, Lee SJ, Kim CW, Kim SC, Kim SG (2006). Liquiritigenin, an aglycone of liquiritin in Glycyrrhizae radix, prevents acute liver injuries in rats induced by acetaminophen with or without buthionine sulfoximine. Chem Biol Interact.

[38] Sareddy GR, Vadlamudi RK (2015). Cancer therapy using natural ligands that target estrogen receptor beta. Chin J Nat Med.

[39] Wang D, Lu J, Liu Y, Meng Q, Xie J, Wang Z, Teng L (2014). Liquiritigenin induces tumor cell death through mitogen-activated protein kinase- (MPAKs-) mediated pathway in hepatocellular carcinoma cells. Biomed Res Int.

[40] Liu Y, Xie S, Wang Y, Luo K, Cai Y (2012). Liquiritigenin inhibits tumor growth and vascularization in a mouse model of HeLa cells. Molecules.

[41] Ghosh RN, DeBiasio R, Hudson CC, Ramer ER, Cowan CL, Oakley RH (2005). Quantitative cell-based high-content screening for vasopressin receptor agonists using transfluor technology. J Biomol Screen.

[42] Zhang JH, Chung TD, Oldenburg KR (1999). A Simple Statistical Parameter for Use in Evaluation and Validation of High Throughput Screening Assays. J Biomol Screen.

[43] Livak KJ, Schmittgen TD (2001). Analysis of relative gene expression data using real-time quantitative PCR and the 2(-Delta Delta C(T)) Method. Methods.

[44] Alvarez-Fischer D, Henze C, Strenzke C, Westrich J, Ferger B, Hoglinger GU, Oertel WH, Hartmann A (2008). Characterization of the striatal 6-OHDA model of Parkinson’s disease in wild type and alpha-synuclein-deleted mice. Exp Neurol.

[45] Jackson-Lewis V, Przedborski S (2007). Protocol for the MPTP mouse model of Parkinson’s disease. Nat Protoc.

