# Supplementary material for: Estrogen receptor activation contributes to RNF146 expression and neuroprotection in Parkinson's disease models
Source: Oncotarget. 2017 Oct 11;8(63):106721–39. doi: 10.18632/oncotarget.21828 (PMC5739769; doi:10.18632/oncotarget.21828)
Supplement: Supplementary file 1 [file oncotarget-08-106721-s001.pdf]

Estrogen receptor activation contributes to RNF146 expression and neuroprotection in Parkinson's disease models

SUPPLEMENTARY MATERIALS

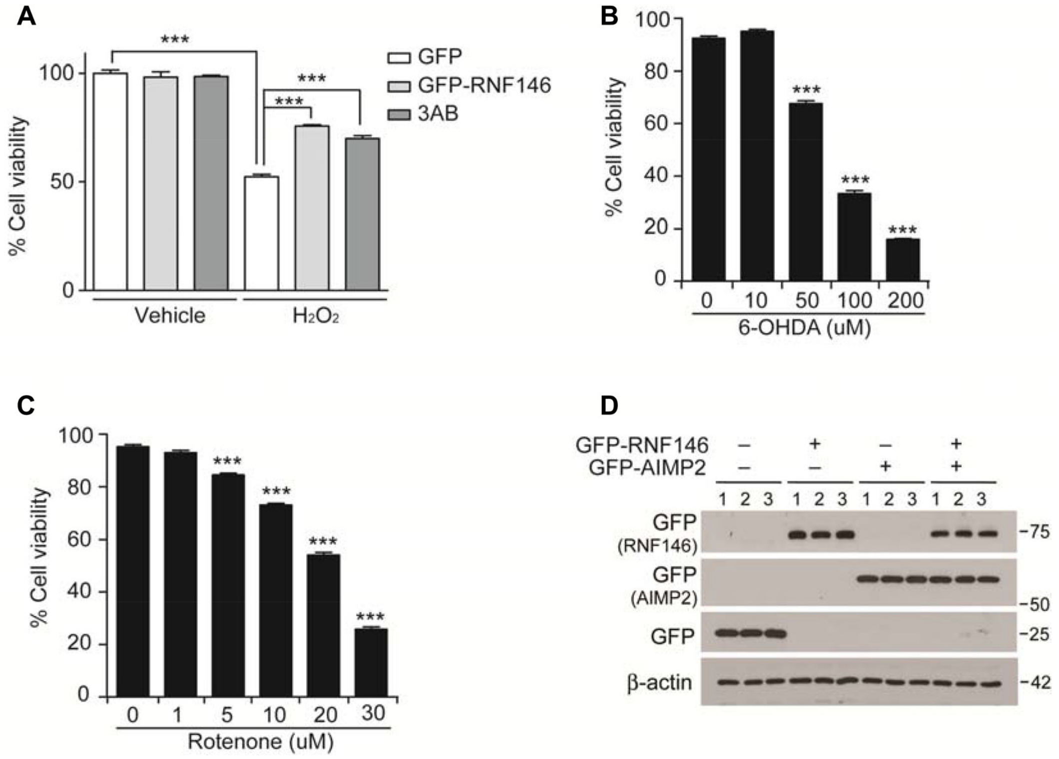

Supplementary Figure 1: (**A**) Cell Counting Kit-8 (CCK8) viability assay demonstrating that GFP-RNF146 expression and PARP inhibition both increase cell survival after stimulation with H2O2 (1 mM). SH-SY5Y cells were transfected with constructs driving the expression of GFP or GFP-RNF146 for 24 hrs and then treated for 24 hrs with H2O2. The PARP inhibitor 3AB (10 uM) was added to SH-SY5Y cells 4 hrs before toxin treatment (*n* = 6). (**B**) Trypan blue exclusion viability assay in SH-SY5Y cells treated with increasing concentrations of 6-OHDA (10, 50, 100, and 200 uM) for 24 hrs (*n* = 6). (**C**) Trypan blue exclusion viability assay in SH-SY5Y cells treated with increasing concentrations of rotenone (1, 5, 10, 20, and 30 uM) for 24 hrs (*n* = 6). (**D**) Expression levels of RNF146 and AIMP2 in SH-SY5Y cells transfected with the indicated combinations of GFP-RNF146 and GFP-AIMP2. Western blotting was carried out using the designated antibodies. β-actin serves as a loading control.

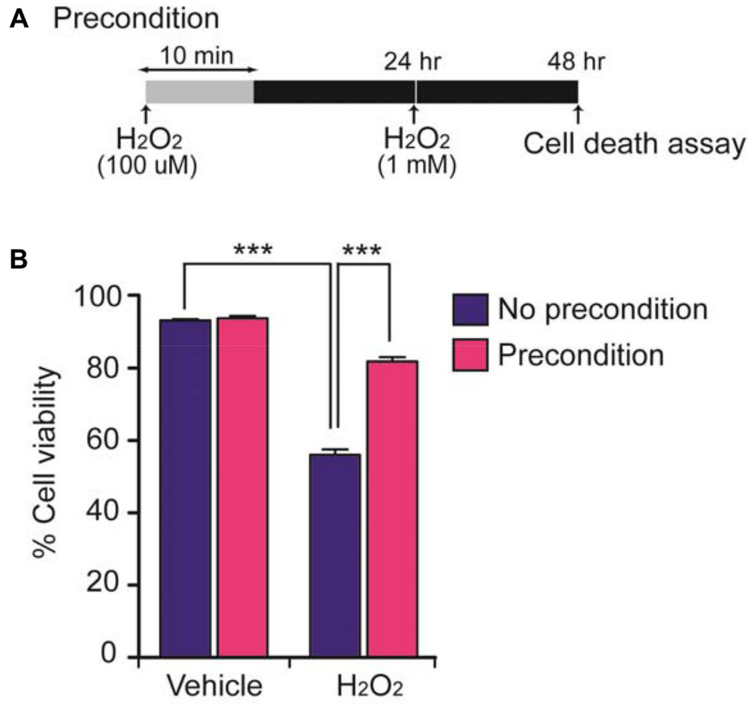

Supplementary Figure 2: (**A**) Schematic illustration of the experimental schedule for H2O2 preconditioning (100 uM, 10 min) in SH-SY5Y cells followed by treatment with toxic concentrations of H2O2 and cell viability assessment. (**B**) Trypan blue exclusion viability assay in SH-SY5Y cells challenged with 1 mM H2O2 for 24 hrs with or without low-dose H2O2 preconditioning ($n = 6$). Data are expressed as mean ± SEM. $^{***}P < 0.001$, ANOVA test followed by Tukey's post hoc analysis.

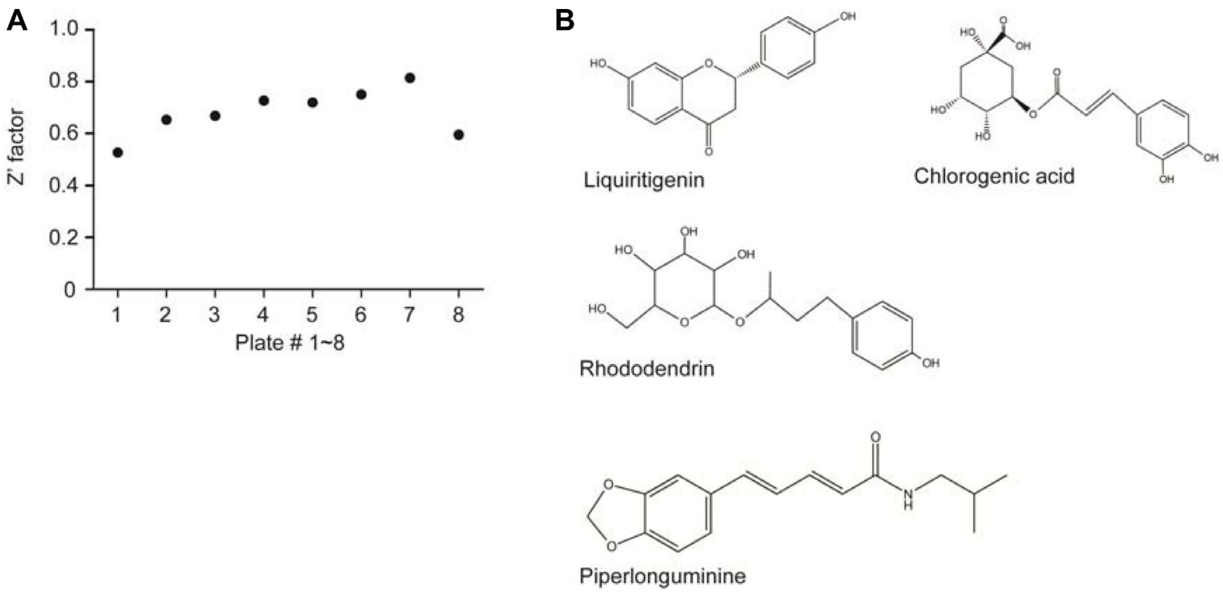

Supplementary Figure 3: (**A**) Scatter plot of the Z' factors of the 96-well plates used in the high-throughput luciferase screen. The Z' factor was calculated for each plate using the means (M) and standard deviations (SD) from the negative (DMSO) and positive (H2O2 preconditioning (100 uM, 10 min)) controls. Z' = 1-3(SDCCCP+SDDMSO)/(MCCCP-MDMSO). (**B**) Chemical structures of the natural compounds (liquiritigenin, chlorogenic acid, rhododendrin, piperlonguminine) that increased RNF146 promoter activity in the luciferase assay.

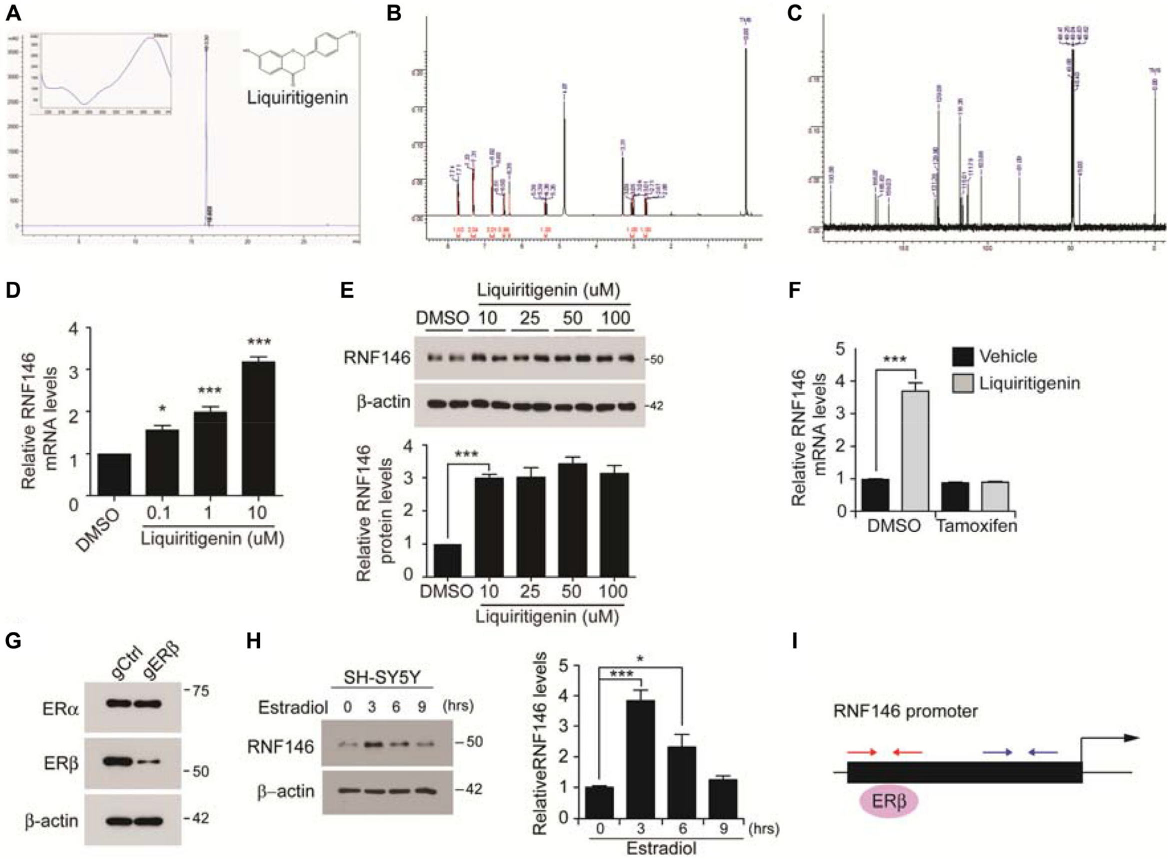

Supplementary Figure 4: (**A**) UV spectrum, HPLC-ELSD spectrum, and chemical structure of liquiritigenin extracted from Glycyrrhiza uralensis. (**B**, **C**) ^{1}H- and ^{13}C-NMR spectra of purified liquiritigenin demonstrating its chemical structure. (**D**) Quantification of relative RNF146 messenger RNA expression in SH-SY5Y cells treated with DMSO or liquiritigenin (37 hrs) at the indicated concentrations. GAPDH was used as an internal loading control (*n* = 3). (**E**) Immunoblot analysis of RNF146 levels in SH-SY5Y cells treated with the indicated concentrations of liquiritigenin for 37 hrs. Quantification of relative RNF146 expression levels (normalized to those of β-actin) from panel A (*n* = 3). (**F**) Quantification of relative RNF146 mRNA levels in SH-SY5Y cells treated with the indicated combinations of liquiritigenin (10 uM, 48 hrs) and tamoxifen (1 uM, 8 hrs). Values are normalized to those of GAPDH (*n* = 3). (**G**) Immunoblots of RNF146 in lysates from SH-SY5Y cells treated with estradiol (10 nM) for the indicated periods of time. β-actin serves as an internal loading control. Quantification of relative RNF146 expression levels. Values are normalized to those of β-actin in panel A (n = 3, bottom panel). (**H**) Specific knockout of ER*α* by CRISPR-cas9 in SH-SY5Y cells determined by western blot using the indicated antibodies. ER*α* was used as a negative control. (**I**) Illustration of RNF146 promoter structure and potential ER*α* binding motif. Approximate binding sites for primer sets are indicated by arrows. Red arrows (F: -1848 to -1828; R: -1620 to -1579 bp from transcription start site of RNF146) for putative ER*α* binding motif; Blue arrows (F: -487 to -445; R: -318 to -299 bp from transcription start site of RNF146) for negative control within RNF146 promoter.

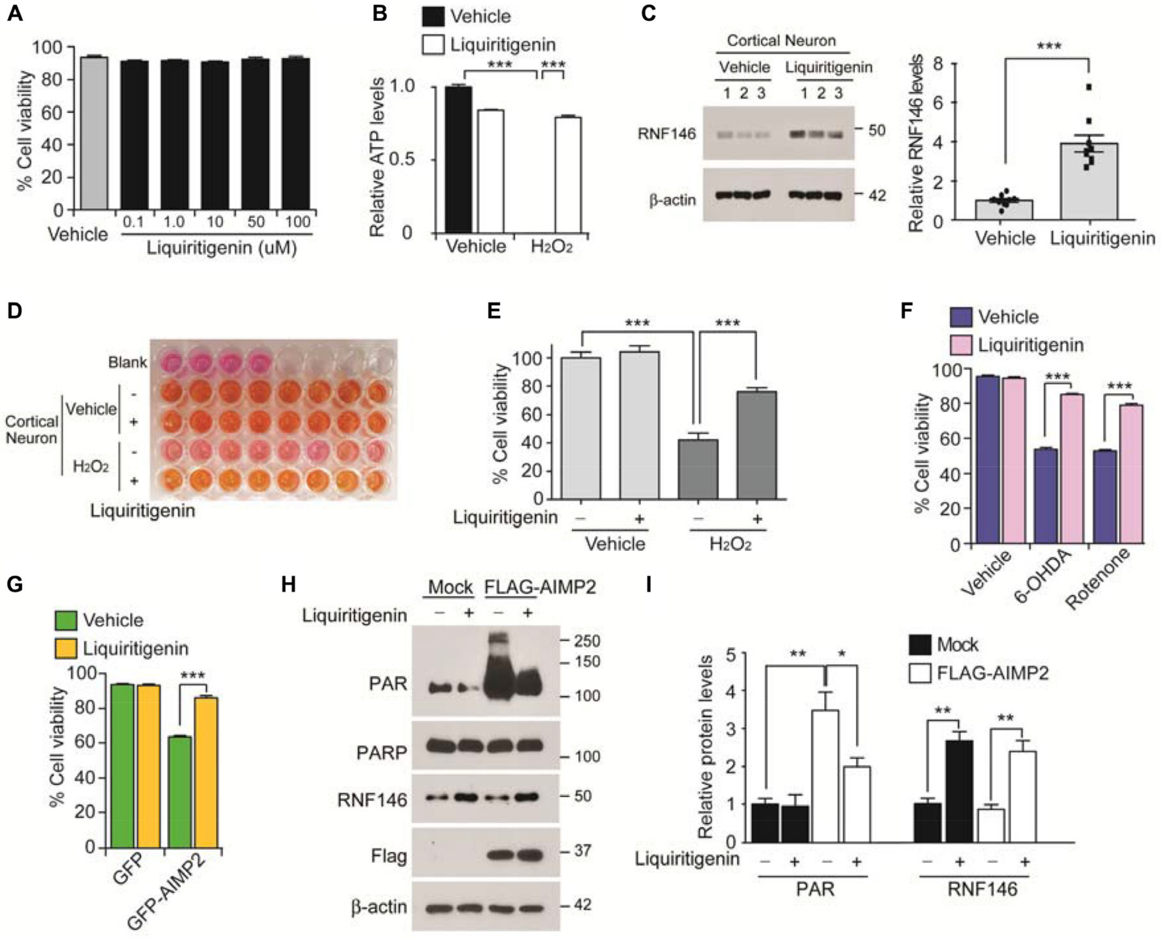

Supplementary Figure 5: (**A**) Trypan blue exclusion viability assay in SH-SY5Y cells treated for 24 hrs with increasing concentrations of liquiritigenin (*n* = 6). (**B**) Assessment of relative intracellular ATP levels in SH-SY5Y cells challenged with H2O2 (1 mM, 24 hrs) following pretreatment with liquiritigenin (10 uM, 48 hrs) or DMSO vehicle as a control (*n* = 6). (**C**) Expression of RNF146 in mouse primary cortical neuron culture treated with DMSO or liquiritigenin determined by western blots. Quantification of relative RNF146 expression in liquiritigenin (10 uM, 37 hrs) treated group as compared to DMSO control (*n* = 9, right panel). (**D**) CCK8 viability assay in mouse cortical neurons pretreated with liquiritigenin (10 uM, 37 hrs) and challenged with H2O2 (100 uM, 24 hrs). (**E**) Spectrophotometry analysis of CCK8 viability assay for panel D (*n* = 8 per group). (**F**) Trypan blue exclusion viability assay in SH-SY5Y cells demonstrating the protective effect of liquiritigenin (10 uM, 48 hrs) against 6-OHDA (70 uM, 24 hrs) and rotenone (20 uM, 24 hrs)-induced cell death (*n* = 6). (**G**) Trypan blue exclusion viability assay in SH-SY5Y cells demonstrating the protective effect of liquiritigenin (10 uM) against AIMP2 overexpression (30 hrs) (*n* = 6). (**H**) Immunoblots of PAR, PARP1, RNF146, and FLAG-AIMP2 in lysates from SH-SY5Y cells transiently transfected with FLAG-AIMP2 or mock (60 hrs) and treated with liquiritigenin (10 uM, 37 hrs). β-actin serves as an internal loading control. (**I**) Quantification of relative PAR-conjugated proteins and RNF146 expression levels in the indicated experimental groups. Values are normalized to those of β-actin (*n* = 3).

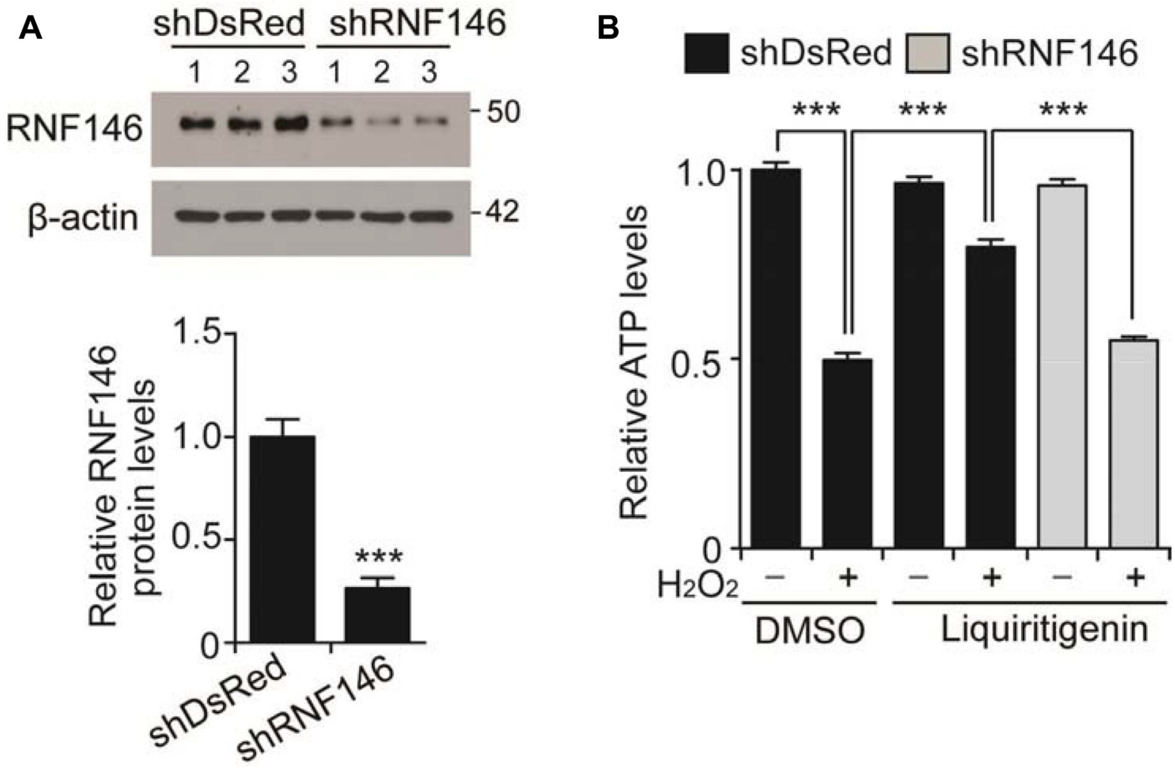

Supplementary Figure 6: (**A**) Representative western blots of RNF146 expression in SH-SY5Y cells transfected with shRNA targeting RNF146 or DsRed as a control. β-actin serves as a loading control. Quantification of relative RNF146 expression in SH-SY5Y cells transfected with shRNA targeting RNF146 or DsRed as a control (*n* = 3, bottom panel). Values are normalized to those of β-actin. (**B**) Assessment of relative intracellular ATP levels in SH-SY5Y cells transfected with shRNA directed against RNF146 (64 hrs, *n* = 6) and challenged with H2O2 (1 mM, 16 hrs) with or without pretreatment with liquiritigenin (10 uM, 40 hrs) or DMSO vehicle. shDsRed was used as an shRNA transfection control.

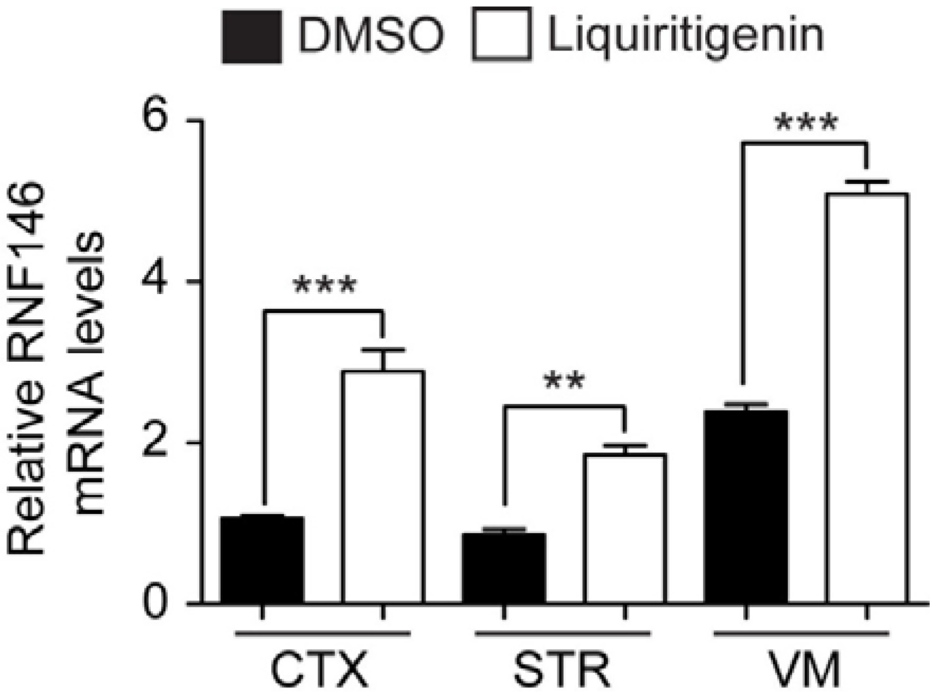

Supplementary Figure 7: Relative expression levels of RNF146 mRNA in the indicated brain subregions from 2-month-old mice treated with DMSO or liquiritigenin (i.p., 10 mg/kg/day, 3 days) determined by realtime quantitative PCR using specific primers for mouse RNF146 (*n* = 4 mice). GAPDH was used as an internal loading control.
